# Biological and Targeted Therapies in the Multidisciplinary Management of Gastrointestinal Cancers

**DOI:** 10.3390/cancers18162675

**Published:** 2026-08-18

**Authors:** Marek Kos, Krzysztof Bojarski, Milena Czosnek, Jan Śnieżyński, Bartosz Wilczyński, Paulina Mertowska, Ewelina Grywalska, Sebastian Mertowski

**Affiliations:** 1 Department of Epidemiology, Institute of Rural Health, 20-090 Lublin, Poland; 2 General Surgery Department, Independent Public Health Care Facility in Łęczna (SP ZOZ in Łęczna), 21-010 Łęczna, Poland; 3 Department of Experimental Immunology, Medical University of Lublin, 20-093 Lublin, Polandpaulina.mertowska@umlub.edu.pl (P.M.); ewelina.grywalska@umlub.edu.pl (E.G.); 4 Student Research Group of Experimental Immunology, Medical University of Lublin, 20-093 Lublin, Poland; 5 Doctoral School of the Medical University of Lublin, Medical University of Lublin, 20-093 Lublin, Poland; 6 II Clinic of General, Gastroenterological and Digestive System Cancer Surgery, University Clinical Hospital No. 1, 20-081 Lublin, Poland

**Keywords:** gastrointestinal cancers, targeted therapy, biological therapy, immunotherapy, immune checkpoint inhibitors, predictive biomarkers, precision oncology, surgery

## Abstract

Gastrointestinal (GI) cancers represent a diverse group of malignancies that remain a major cause of cancer-related morbidity and mortality worldwide. Their management is increasingly complex, reflecting differences in tumor biology, anatomical location, stage, and molecular profile. In recent years, advances in molecular diagnostics, immunotherapy, and targeted treatment have moved clinical decision-making beyond a purely organ- and stage-based approach toward more individualized, biomarker-guided care. This narrative review summarizes established and emerging biological and targeted therapies used in esophageal, gastric and gastroesophageal junction, colorectal, pancreatic, hepatocellular, and biliary tract cancers. It focuses on immune checkpoint inhibitors targeting PD-1, PD-L1, and CTLA-4; HER2-directed monoclonal antibodies and antibody–drug conjugates; antiangiogenic and anti-EGFR therapies; and newer strategies involving CLDN18.2, FGFR2b, and tumor-agnostic alterations such as NTRK fusions. The review also considers the predictive biomarkers used to guide treatment selection and the growing integration of systemic therapy with surgery in neoadjuvant, perioperative, adjuvant, and conversion settings. However, clinical efficacy alone does not determine whether new treatments become part of routine practice. Regulatory approval, reimbursement, access to molecular testing, and the availability of specialized multidisciplinary care are equally important. The rapidly evolving treatment landscape for GI cancers therefore requires clinical decisions that account for tumor biology, anatomical resectability, molecular eligibility, expected benefit, treatment-related toxicity, and local access to therapy. Expanding access to comprehensive biomarker testing and effective molecularly guided treatments will be essential to translate progress in precision oncology into more personalized and equitable care for patients with GI cancers.

## Simple Summary

Gastrointestinal cancers are among the most common and deadly cancers worldwide. Their treatment is becoming increasingly complex because many new therapies no longer depend only on the organ in which the tumor develops, but also on specific biological features of the cancer cells. This review explains how modern biological and targeted therapies work in gastrointestinal cancers, which biomarkers help identify patients who may benefit from them, and how these treatments can be combined with surgery, chemotherapy, radiotherapy, or immunotherapy. The aim is to provide a clear overview of current and emerging treatment strategies, including immune checkpoint inhibitors, anti-HER2 therapies, antiangiogenic drugs, anti-EGFR antibodies, and newer approaches targeting CLDN18.2 and FGFR2b. By summarizing mechanisms, biomarkers, and clinical use, this review may help researchers and clinicians better understand the changing landscape of personalized treatment for gastrointestinal cancers.

## 1. Introduction

Gastrointestinal (GI) cancers constitute a heterogeneous group of malignancies that differ substantially in their anatomical origin, histological characteristics, molecular landscape, clinical course, and sensitivity to treatment. They encompass a heterogeneous group of clinicopathological entities, including esophageal cancer, gastric and gastroesophageal junction cancer, colorectal cancer, pancreatic cancer, hepatocellular carcinoma, and biliary tract cancer [1,2,3]. According to Global Cancer Observatory (GLOBOCAN) 2022 data, nearly 20 million new cancer cases and approximately 9.7 million cancer-related deaths were recorded worldwide [4]. GI cancers represent a particularly significant portion of this burden, accounting globally for approximately one-quarter of new cancer cases and one-third of cancer deaths; for the five main GI sites—colorectal, stomach, liver, esophagus, and pancreas—approximately 4.8 million cases and 3.4 million deaths were estimated. Projections further indicate that by 2050, the global number of new GI cancer cases could increase to approximately 9.06 million, and the number of deaths to 6.42 million. The high mortality rate of many GI cancers is due to late diagnosis, aggressive tumor biology, the ability to infiltrate and metastasize early, and limited treatment efficacy in some patients with advanced disease [3,4,5]. For many years, treatment strategies for GI cancers were determined predominantly by tumor location, histological type, clinical stage, and anatomical resectability. Surgery remains the principal curative-intent treatment for most localized GI malignancies, whereas chemotherapy and radiotherapy continue to play important roles in neoadjuvant, perioperative, adjuvant, and palliative settings. However, contemporary management increasingly requires the coordinated use of surgery, systemic therapy, radiotherapy, locoregional interventions, and molecular diagnostics within multidisciplinary treatment pathways. Consequently, therapeutic decisions are no longer based solely on whether a tumor can be technically resected, but also on its biological characteristics, predicted sensitivity to systemic treatment, potential for downstaging or conversion to resectability, and the risks associated with perioperative therapy [6,7,8].

Advances in molecular profiling, next-generation sequencing (NGS), immuno-oncology and biomarker-based drug development have transformed the therapeutic landscape of GI cancers. Clinically relevant biomarkers now include microsatellite instability-high (MSI-H) and deficient mismatch repair (dMMR) status, programmed death-ligand 1 (PD-L1) expression, human epidermal growth factor receptor 2 (HER2) status, mutations in *KRAS*, *NRAS*, and *BRAF*, neurotrophic tyrosine receptor kinase (*NTRK*) gene fusions, claudin 18.2 (CLDN18.2) expression, fibroblast growth factor receptor 2 (*FGFR2*) alterations, isocitrate dehydrogenase 1 (*IDH1*) mutations, and defects in homologous recombination repair (HRR), including alterations in *BRCA1*, *BRCA2*, and *PALB2* [9,10,11].

These biomarkers may identify patients eligible for immune checkpoint inhibitors (ICIs), monoclonal antibodies (mAbs), antibody–drug conjugates (ADCs), antiangiogenic agents, tyrosine kinase inhibitors (TKIs), and other molecularly targeted treatments. The introduction of tumor-agnostic therapies has further challenged the traditional organ-based model by allowing treatment selection according to a specific molecular alteration rather than the primary tumor site [12,13].

Despite this progress, several clinically important issues are often discussed separately. Reviews focusing on molecular mechanisms may provide limited information on how targeted and biological therapies alter surgical decision-making. Conversely, clinically oriented reviews may summarize treatment recommendations without critically integrating the biological basis of patient selection, the limitations of predictive biomarkers, or the discrepancy between regulatory approval and actual access to treatment. In addition, the presence of an actionable biomarker and the authorization of a drug by the United States Food and Drug Administration (FDA) or the European Medicines Agency (EMA) do not necessarily translate into routine clinical availability. Reimbursement rules, access to validated molecular testing, treatment-line restrictions, and national funding criteria may substantially limit the implementation of precision oncology in real-world practice.

Another unresolved challenge concerns the interpretation of predictive biomarkers across different GI malignancies. MSI-H and dMMR status represent robust predictors of benefit from immune checkpoint blockade in selected clinical settings, whereas the predictive value of PD-L1 expression varies according to tumor type, histological subtype, scoring system, treatment regimen, and line of therapy. PD-L1 expression may be assessed using different scoring approaches, including the combined positive score (CPS) and tumor proportion score (TPS), which are not interchangeable and have different clinical relevance depending on the cancer type and therapeutic regimen. Similarly, the clinical relevance of HER2, *KRAS*, *NRAS*, *BRAF*, CLDN18.2, *FGFR2*, *IDH1*, *BRCA1*, *BRCA2*, *PALB2*, *KRAS* G12C, and *NTRK* alterations depends on the disease context and the availability of matched therapies. Therefore, a comprehensive assessment of modern GI oncology requires not only the identification of therapeutic targets, but also critical evaluation of biomarker validity, treatment setting, surgical implications, regulatory status, and practical accessibility.

The aim of this narrative review is to critically synthesize current and emerging biological and molecularly targeted therapies in major GI cancers. The review focuses on four interconnected dimensions: the mechanisms of action of selected therapeutic classes; the predictive biomarkers used for patient selection; the influence of systemic therapies on neoadjuvant, perioperative, adjuvant, conversion, and secondary resection strategies; and the distinction between regulatory authorization and real-world therapeutic availability. By integrating molecular oncology, multidisciplinary surgical decision-making, and treatment accessibility, this review aims to provide a clinically oriented framework for interpreting the rapidly evolving landscape of precision therapy in GI cancers.

## 2. Materials and Methods

### 2.1. Review Design and Scope

This study was designed as a narrative review of biological and molecularly targeted therapies used or investigated in GI cancers. The review was conducted in accordance with the general principles of methodological transparency for narrative reviews and was structured with reference to the Scale for the Assessment of Narrative Review Articles (SANRA) [14]. Owing to the narrative character of the study, no formal meta-analysis, quantitative evidence synthesis, or systematic risk-of-bias assessment was performed.

The review focused on esophageal cancer, gastric cancer (GC), gastroesophageal junction (GEJ) cancer, colorectal cancer (CRC), pancreatic ductal adenocarcinoma (PDAC), hepatocellular carcinoma (HCC), and biliary tract cancer (BTC). Anal cancer, gastrointestinal stromal tumors, neuroendocrine tumors, and selected rare GI malignancies were considered only when they illustrated clinically relevant mechanisms of action, biomarker-driven treatment, or tumor-agnostic therapeutic strategies. The main areas of analysis included the mechanisms of action of biological and targeted therapies, predictive biomarkers used for patient selection, integration of systemic therapies with surgical treatment, regulatory authorization by the United States Food and Drug Administration (FDA) and the European Medicines Agency (EMA), and therapeutic accessibility within the Polish reimbursement system.

### 2.2. Literature Search Strategy

A structured literature search was conducted using the PubMed and Web of Science databases. No restriction on publication date was applied, allowing the inclusion of both seminal publications and recent evidence relevant to the development and current clinical application of biological and targeted therapies in GI cancers. The search strategy combined terms related to GI malignancies with terms describing biological therapies, molecularly targeted treatments, predictive biomarkers, precision oncology, and surgical integration. The principal search terms included combinations of the following keywords and Medical Subject Headings, where applicable: “gastrointestinal cancer,” “gastrointestinal malignancy,” “esophageal cancer,” “gastric cancer,” “gastroesophageal junction cancer,” “colorectal cancer,” “pancreatic cancer,” “hepatocellular carcinoma,” “biliary tract cancer,” “cholangiocarcinoma,” “biological therapy,” “targeted therapy,” “precision oncology,” “predictive biomarker,” “immunotherapy,” “immune checkpoint inhibitor,” “PD-1,” “PD-L1,” “CTLA-4,” “MSI-H,” “dMMR,” “HER2,” “EGFR,” “VEGF,” “VEGFR,” “CLDN18.2,” “FGFR2,” “IDH1,” “BRAF,” “KRAS G12C,” “BRCA,” “PARP inhibitor,” “NTRK fusion,” “tumor-agnostic therapy,” “neoadjuvant treatment,” “perioperative treatment,” “conversion therapy,” “secondary resection,” and “surgery.”

Boolean operators “AND” and “OR” were used to combine terms describing individual tumor types, therapeutic targets, biomarkers, treatment settings, and surgical strategies. Additional publications were identified by manually screening the reference lists of relevant reviews, clinical guidelines, pivotal clinical trials, and regulatory documents.

### 2.3. Study Selection, Data Synthesis, and Assessment of Regulatory and Reimbursement Status

Publications were eligible if they addressed the mechanisms, biomarkers, clinical application, safety, or surgical integration of biological and molecularly targeted therapies in GI cancers. Studies concerning regulatory approval, reimbursement, precision oncology, molecular profiling, liquid biopsy, and longitudinal biomarker monitoring were also considered. Priority was given to clinical guidelines, official regulatory documents, pivotal clinical trials, prospective studies, meta-analyses, systematic reviews, and high-quality narrative reviews. Relevant preclinical and translational studies were included when necessary to explain a therapeutic mechanism, support the biological rationale for a biomarker, or describe an emerging target.

Studies unrelated to GI malignancies or targeted treatment, reports of limited methodological or clinical relevance, non-informative abstracts, and duplicate publications were excluded. Conventional chemotherapy and radiotherapy were considered only when discussed as part of combined biological, targeted, or surgical treatment strategies. Titles and abstracts were screened for relevance, followed by full-text assessment of potentially eligible publications. The search was limited to English-language articles, and disagreements regarding study inclusion were resolved by consensus between the authors. Data were synthesized according to cancer type, therapeutic class, molecular target, mechanism of action, predictive biomarker, clinical application, relationship with surgical management, regulatory status, and therapeutic availability in Poland. The evidence was organized primarily by therapeutic mechanism and clinical relevance, with particular attention to differences in biomarker significance across GI cancers and to the distinction between established and investigational therapies. Regulatory status was assessed using official sources from the United States Food and Drug Administration (FDA) and the European Medicines Agency (EMA) [15,16]. For each therapy, it was determined whether the medicinal product had received marketing authorization from the respective agency. This assessment was general and did not constitute a detailed analysis of indication-specific approval, treatment line, biomarker requirements, or authorization conditions.

Therapeutic accessibility in Poland was assessed using official reimbursement documents published by the Polish Ministry of Health, including reimbursement lists [17]. The analysis was based on documents valid as of 1 January 2026 and focused on whether a medicinal product was present on the analyzed reimbursement lists. The presence of a drug on such a list was not interpreted as confirmation of reimbursement for every registered GI cancer indication. Therefore, the results reflect general listing status rather than indication-specific funding conditions. Because regulatory and reimbursement information may change over time, the reported status should be interpreted as applicable only to the defined assessment date.

## 3. Results

### 3.1. Clinical and Molecular Landscape of GI Cancers

GI cancers comprise a heterogeneous group of malignancies that differ substantially in anatomical origin, predominant histology, molecular background, biological behavior, metastatic pattern, and sensitivity to treatment. The principal entities discussed in this review include esophageal cancer, GC and GEJ cancer, CRC, PDAC, HCC, and BTCs. Selected rarer malignancies, including anal cancer, small bowel cancer, gastrointestinal stromal tumors (GISTs), and neuroendocrine neoplasms, are also considered because they illustrate distinct mechanisms of carcinogenesis and biomarker-dependent treatment [3,11,18]. The extent to which molecular and immunological features influence clinical decision-making varies considerably across these malignancies. In some GI cancers, molecular stratification is routinely used to guide treatment selection, whereas in others it remains restricted to selected subgroups or has predominantly prognostic and biological relevance. The role of surgery and multimodal treatment also differs according to tumor type, disease stage, anatomical resectability, and the availability of effective systemic therapies. Table 1 provides a comparative overview of these clinicopathological, molecular, and therapeutic differences and highlights the unequal maturity of biomarker-guided treatment across GI oncology [18,19,20,21,22,23,24,25,26,27,28,29,30,31,32,33,34,35,36,37,38,39,40,41,42,43].

### 3.2. Biologics and Targeted Therapies by Mechanism of Action

Biological and molecularly targeted therapies have expanded treatment options for selected patients with GI cancers by interfering with specific immune checkpoints, receptors, ligands, signaling pathways, or molecular drivers of tumor growth. Unlike conventional cytotoxic chemotherapy, these strategies require increasingly precise patient selection based on tumor type, disease setting, molecular profile, and the availability of a validated predictive biomarker. Their clinical relevance therefore differs substantially across individual GI malignancies, ranging from established standards of care to treatments restricted to small molecular subgroups or ongoing clinical trials [11,44,45].

#### 3.2.1. Immune Checkpoint Inhibitors

ICIs have transformed the treatment of several GI cancers by restoring antitumor T-cell activity. The PD-1/PD-L1 and CTLA-4 pathways regulate different stages of the immune response. CTLA-4 primarily limits early T-cell activation by competing with CD28 for binding to CD80 and CD86 on APCs. In contrast, PD-1 signaling acts mainly during the effector phase. Binding of PD-1 to PD-L1 or PD-L2 suppresses T-cell proliferation, cytokine production, and cytotoxic activity within the TME. Tumor and immune cells may exploit these inhibitory pathways to promote immune escape and tumor survival [46,47,48,49].

**Figure 1:**
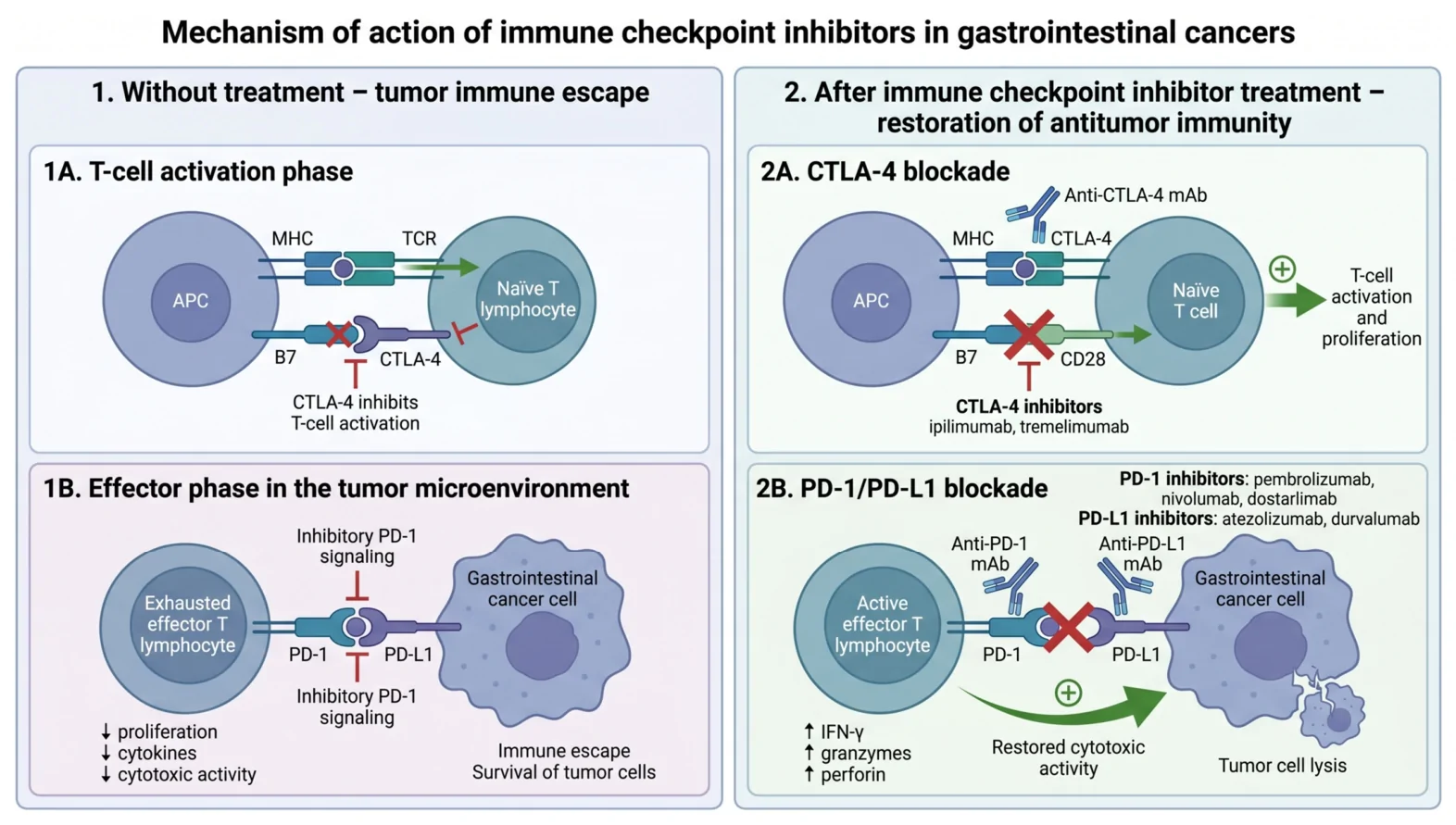
Mechanisms of immune checkpoint blockade in GI cancers [23,47,48,49,50,51,52]. During T-cell priming, CTLA-4 competes with CD28 for binding to B7 ligands on APCs, thereby limiting costimulation and T-cell activation. In the TME, PD-1/PD-L1 signaling promotes effector T-cell dysfunction, reduced cytokine production, impaired cytotoxicity, and tumor immune escape. CTLA-4 blockade restores CD28-mediated costimulation and promotes T-cell activation and proliferation, whereas PD-1 or PD-L1 blockade restores effector T-cell activity, enhances the release of IFN-γ, granzymes, and perforin, and promotes tumor cell lysis. Created in BioRender. Mertowski, S. (2026) https://BioRender.com/wgsbmpm (accessed on 12 August 2026).

Blockade of PD-1 or PD-L1 interrupts inhibitory signaling and may restore effector T-cell function, whereas CTLA-4 blockade enhances T-cell priming and costimulation. Combined inhibition of these pathways may produce complementary immune activation, but it is also associated with a higher incidence of irAEs than monotherapy [48,49,50,51,52]. The principal mechanisms of tumor immune escape and their reversal by ICIs are summarized in Figure 1.

MSI-H/dMMR is the most consistently validated predictive biomarker of response to immune checkpoint blockade in GI oncology. Defective MMR promotes the accumulation of somatic mutations and neoantigens, thereby increasing tumor immunogenicity [53,54]. The approval of pembrolizumab for previously treated, unresectable or metastatic MSI-H/dMMR solid tumors marked a major step toward tumor-agnostic therapy [55]. In CRC, MSI-H/dMMR is routinely used to identify candidates for immunotherapy, whereas most MSS tumors remain poorly responsive to monotherapy with PD-1/PD-L1 inhibitors; combination strategies aimed at overcoming this resistance are still largely investigational [55,56,57,58].

The predictive value of PD-L1 is less uniform and depends on tumor type, histology, assay, scoring system, treatment regimen, and line of therapy. In GC, GEJ cancer, and ESCC, PD-L1 is commonly assessed using CPS, although clinically relevant thresholds vary between indications. Accordingly, PD-L1 should be interpreted within the context of the specific therapeutic setting rather than as an independent universal biomarker [59,60,61,62].

In HCC, ICIs are used mainly combined with antiangiogenic therapy or CTLA-4 blockade, despite the absence of a routinely validated predictive biomarker [63,64]. In advanced BTCs, checkpoint inhibition is primarily combined with gemcitabine and cisplatin, while MSI-H/dMMR may identify a rare subgroup with particularly high sensitivity [46,65,66]. By contrast, PDAC remains largely resistant to immunotherapy because of its low immunogenicity, dense stroma, limited T-cell infiltration, and immunosuppressive TME; ICIs currently have an established role mainly in the rare MSI-H/dMMR subgroup [67,68]. CTLA-4 inhibitors, including ipilimumab and tremelimumab, are used more often in combination with PD-1 or PD-L1 blockade than as monotherapy. Although this approach may enhance antitumor activity through complementary immune mechanisms, it also increases the risk of irAEs [48,51,52].

#### 3.2.2. HER2-Targeted Therapies and Antibody–Drug Conjugates

**Figure 2:**
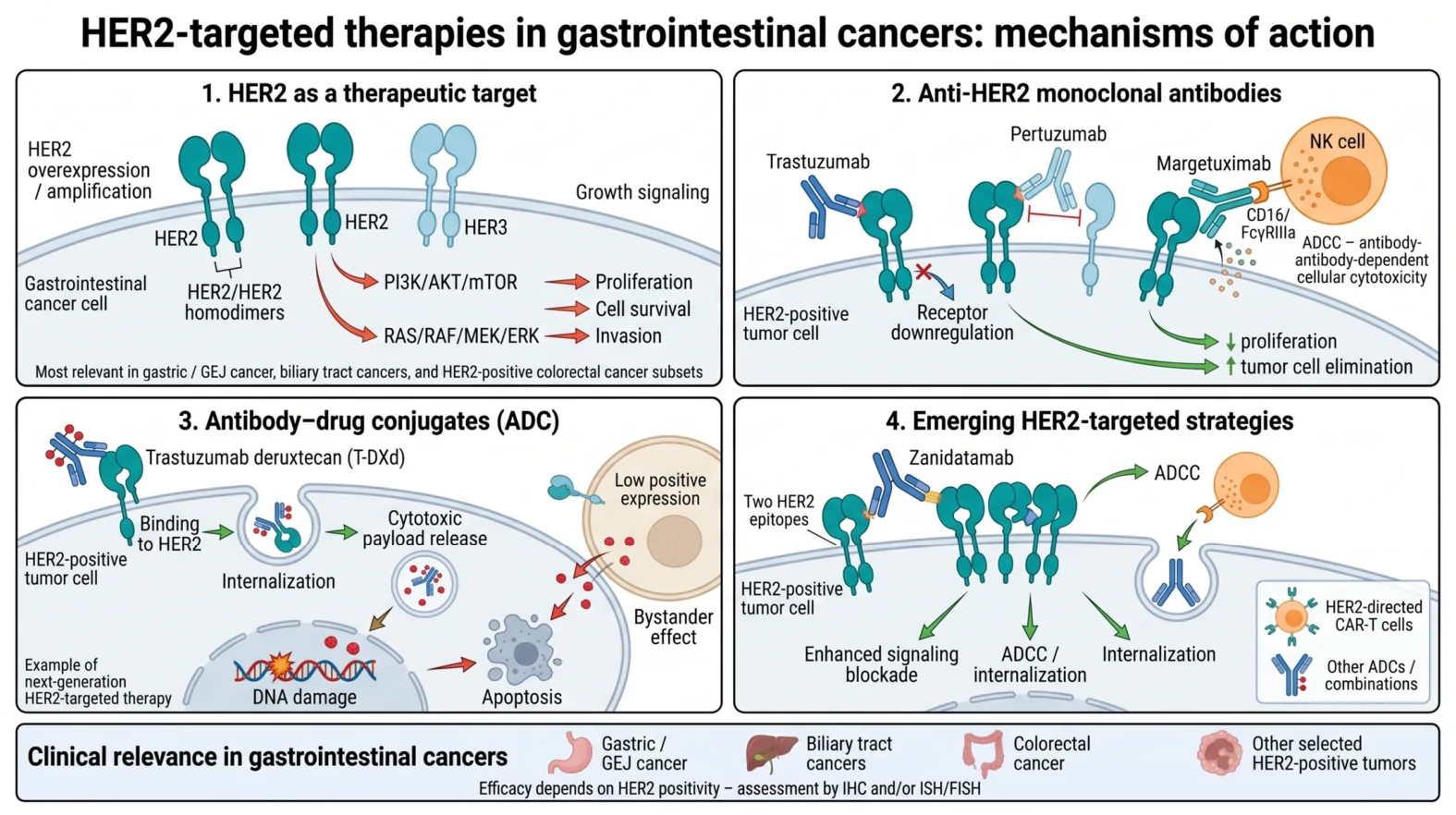
Mechanisms of HER2-directed therapies in GI cancers [69,70,71,72]. HER2 overexpression or *ERBB2* amplification promotes receptor homo- or heterodimerization and activates the PI3K/AKT/mTOR and RAS/RAF/MEK/ERK pathways. Anti-HER2 mAbs inhibit receptor signaling, interfere with receptor dimerization, and may promote ADCC. ADCs deliver cytotoxic payloads into HER2-expressing cells, resulting in DNA damage, apoptosis, and a bystander effect. Emerging strategies include bispecific antibodies, additional ADCs, and HER2-directed cellular therapies. Created in BioRender. Mertowski, S. (2026) https://BioRender.com/wgsbmpm (accessed on 12 August 2026).

HER2, encoded by the *ERBB2* gene, is a member of the epidermal growth factor receptor family and regulates cell proliferation, survival, differentiation, and migration. HER2 overexpression or amplification promotes receptor dimerization and persistent activation of the PI3K/AKT/mTOR and RAS/RAF/MEK/ERK pathways, thereby supporting tumor growth, invasion, resistance to apoptosis, and metastatic progression. In GI oncology, HER2 has the greatest established clinical relevance in GC and GEJ adenocarcinomas, although HER2-directed strategies are also increasingly used or investigated in selected CRC and BTC subgroups (Figure 2) [69,70,71,72].

HER2 status is assessed primarily by immunohistochemistry (IHC), with in situ hybridization (ISH) used to confirm *ERBB2* amplification in equivocal cases. Interpretation in GC and GEJ cancer differs from that used in breast cancer because HER2 expression is often heterogeneous and may show incomplete basolateral or lateral membranous staining. These features increase the risk of sampling error and misclassification, which may lead to either unnecessary treatment or the exclusion of patients who could benefit from HER2-directed therapy [73,74,75,76,77].

Trastuzumab remains the established HER2-directed mAb in HER2-positive GC and GEJ cancer. It inhibits HER2-dependent signaling, promotes receptor internalization, and contributes to ADCC. The development of ADCs has extended this therapeutic concept by combining target recognition with intracellular delivery of a cytotoxic payload. Trastuzumab deruxtecan consists of an anti-HER2 antibody linked to a topoisomerase I inhibitor. Following HER2 binding and internalization, the payload is released within the tumor cell, inducing DNA damage and apoptosis. Its membrane-permeable payload may also produce a bystander effect, which is particularly relevant in tumors with heterogeneous HER2 expression [78,79,80,81].

The clinical role of HER2-directed therapy differs across GI malignancies. In GC and GEJ cancer, HER2 testing is routinely incorporated into treatment selection, and trastuzumab-based therapy remains a central component of treatment in HER2-positive disease. Trastuzumab deruxtecan has further expanded treatment options in previously treated advanced HER2-positive GC and GEJ cancer [82,83,84]. In CRC and BTC, HER2-directed treatment is generally limited to selected molecularly defined subgroups and depends on the level of amplification or overexpression, previous therapy, and the specific agent used.

Emerging strategies include zanidatamab, a bispecific antibody that binds two distinct HER2 epitopes, and additional ADCs such as disitamab vedotin. These approaches are particularly relevant in HER2-positive BTC, GC/GEJ cancer, and selected CRC populations [7,85,86,87]. Pertuzumab-based combinations, margetuximab, HER2-directed cellular therapies, and novel bispecific or multispecific constructs remain investigational or have limited indication-specific roles.

#### 3.2.3. Antiangiogenic Therapies Targeting VEGF and VEGFR

**Figure 3:**
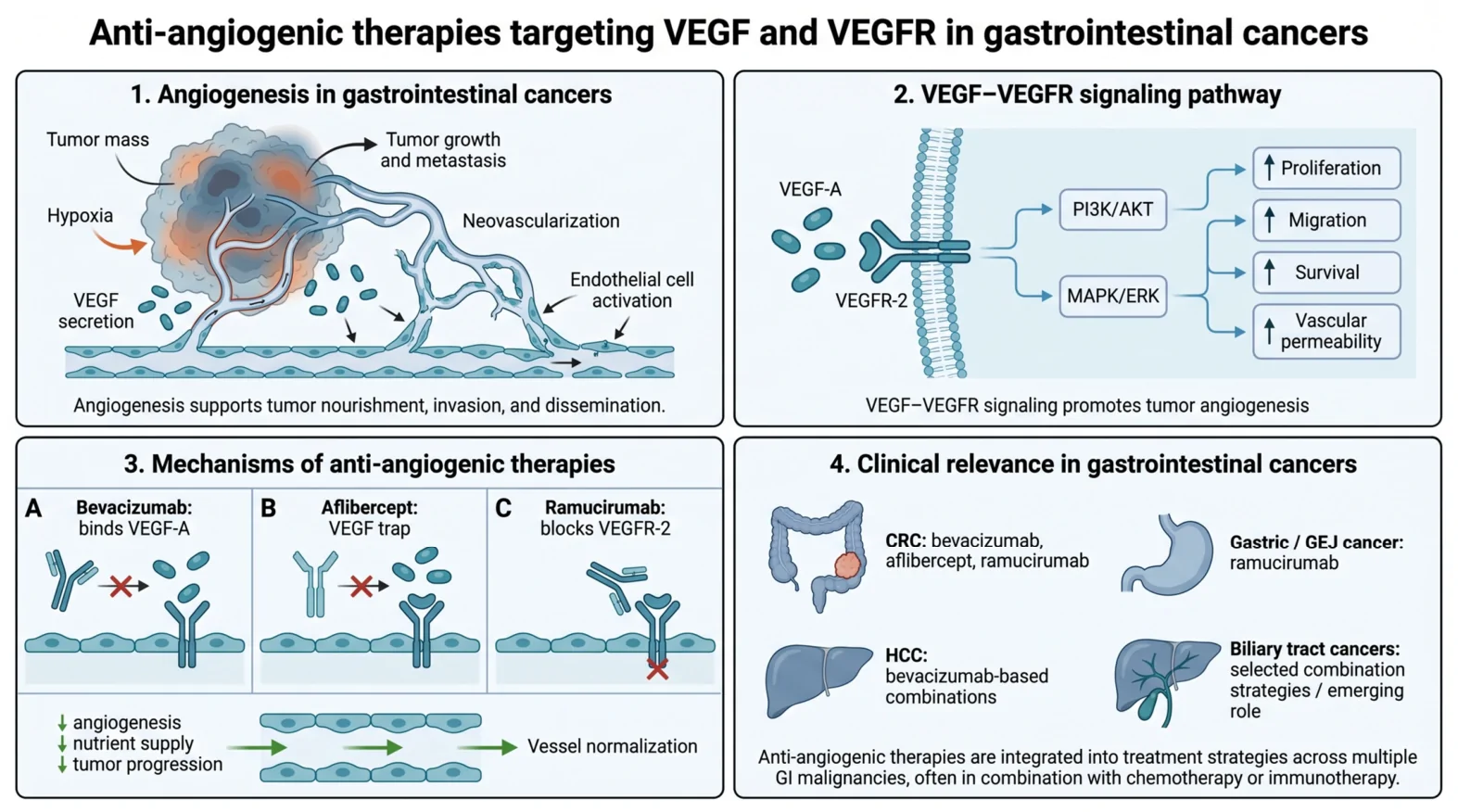
Mechanisms of antiangiogenic therapies targeting VEGF and VEGFR in GI cancers [87,88,89,90]. Tumor hypoxia induces VEGF secretion, leading to VEGFR-2 activation on endothelial cells and stimulation of the PI3K/AKT and MAPK/ERK pathways. This promotes endothelial-cell proliferation, migration, survival, vascular permeability, and abnormal neovascularization. Bevacizumab neutralizes VEGF-A, aflibercept acts as a soluble VEGF-trap, and ramucirumab blocks VEGFR-2 activation. These mechanisms reduce angiogenesis and tumor vascular support and may contribute to vascular normalization. Created in BioRender. Mertowski, S. (2026) https://BioRender.com/wgsbmpm (accessed on 12 August 2026).

Angiogenesis is essential for tumor growth, invasion, and metastatic dissemination by providing oxygen and nutrients and supporting the formation of an abnormal vascular network. The VEGF/VEGFR axis is a major regulator of this process, particularly through VEGF-A-mediated activation of VEGFR-2 on endothelial cells (Figure 3) [88,89,90].

Unlike many biomarker-directed therapies, antiangiogenic treatment is not routinely guided by a single validated predictive biomarker. Treatment selection depends primarily on tumor type, disease setting, line of therapy, prior treatment, patient condition, toxicity profile, and the feasibility of combining antiangiogenic agents with chemotherapy or immunotherapy [91,92]. The principal antiangiogenic agents used in GI oncology include bevacizumab, ramucirumab, and aflibercept. Bevacizumab is a mAb that neutralizes VEGF-A and is widely used in metastatic CRC, as well as in combination with atezolizumab in advanced HCC. Ramucirumab blocks VEGFR-2 and has an established role in selected GC/GEJ cancer settings, as well as in specific CRC and HCC indications. Aflibercept is a soluble decoy receptor that binds VEGF-A, VEGF-B, and PlGF and is used primarily in metastatic CRC [93,94,95,96,97].

These agents illustrate that targeted therapy may act not only against a tumor-specific molecular alteration, but also against a biological process shared by multiple solid tumors. Their efficacy may result from inhibition of neovascularization, reduced vascular permeability, and partial normalization of the tumor vasculature, which may improve the delivery and activity of other systemic treatments. However, the absence of a routinely validated predictive biomarker remains an important limitation. Antiangiogenic therapy also has relevant perioperative implications because VEGF inhibition may impair wound healing and increase the risk of bleeding or thromboembolic complications. These issues are discussed in greater detail in the section on integration with surgery [93,94,95,96,97].

#### 3.2.4. EGFR-, BRAF-, and KRAS-Directed Strategies in CRC

**Figure 4:**
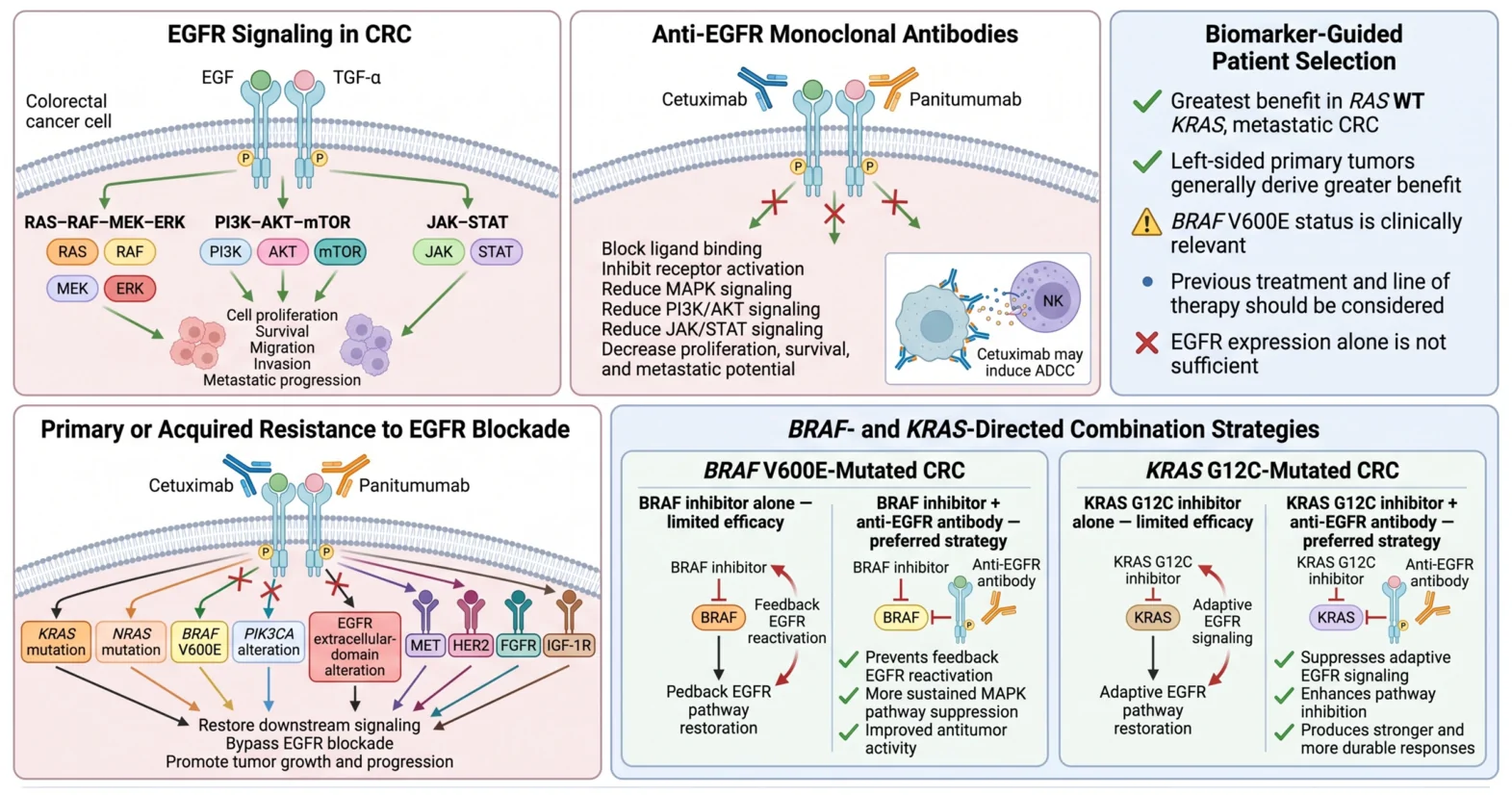
EGFR-, BRAF-, and KRAS-directed therapeutic strategies in CRC [98,99,100,101,102,103,104,105,106,107,108,109,110,111,112,113]. The schematic summarizes EGFR signaling, the mechanisms of anti-EGFR mAbs, biomarker-guided patient selection, and major mechanisms of primary and acquired resistance. Ligand-dependent EGFR activation stimulates the RAS–RAF–MEK–ERK, PI3K–AKT–mTOR, and JAK–STAT pathways, promoting tumor-cell proliferation, survival, migration, invasion, and metastatic progression. Cetuximab and panitumumab block ligand binding and receptor activation, thereby reducing downstream oncogenic signaling; cetuximab may additionally induce ADCC. Clinical benefit is greatest in RAS WT metastatic CRC, particularly in left-sided primary tumors, whereas EGFR expression alone is insufficient for treatment selection. Resistance may arise through alterations in *KRAS*, *NRAS*, *BRAF*, *PIK3CA*, the EGFR extracellular domain, or activation of alternative receptor pathways. In *BRAF* V600E- and *KRAS* G12C-mutated CRC, monotherapy is limited by feedback or adaptive EGFR signaling, supporting combined pathway inhibition with anti-EGFR therapy. Created in BioRender. Mertowski, S. (2026) https://BioRender.com/wgsbmpm (accessed on 12 August 2026).

EGFR regulates epithelial-cell proliferation, survival, migration, and differentiation through downstream pathways including RAS/RAF/MEK/ERK and PI3K/AKT. In GI oncology, anti-EGFR mAbs have their greatest established clinical relevance in metastatic CRC. Cetuximab and panitumumab bind the extracellular domain of EGFR, inhibit ligand-dependent receptor activation, and suppress downstream proliferative signaling. Cetuximab may additionally induce ADCC (Figure 4) [98,99,100,101,102].

The effectiveness of anti-EGFR therapy depends primarily on the absence of activating *KRAS* and *NRAS* mutations. These alterations maintain constitutive downstream signaling despite receptor blockade and therefore function as negative predictive biomarkers. Treatment selection also depends on *BRAF* status, primary tumor sidedness, disease setting, and previous therapy. The greatest benefit is generally observed in patients with left-sided, RAS WT metastatic CRC, whereas activity in right-sided tumors is lower and more context-dependent [102,103,104,105,106,107,108,109].

Resistance to EGFR blockade may be primary or acquired. Primary resistance is commonly associated with activating alterations in *KRAS*, *NRAS*, *BRAF*, or other downstream signaling components. Acquired resistance may develop through the emergence of resistant RAS clones, alterations in the EGFR extracellular domain, activation of alternative receptor pathways, or additional changes affecting the MAPK and PI3K signaling networks. CRC therefore represents a key model in which treatment eligibility is determined not only by the presence of the therapeutic target, but also by the absence of molecular alterations that bypass receptor inhibition [110,111,112,113].

In *BRAF* V600E-mutated metastatic CRC, isolated BRAF inhibition is generally insufficient because feedback reactivation of EGFR restores MAPK signaling. Consequently, treatment strategies combine BRAF inhibition with EGFR blockade, with or without additional pathway inhibition. This approach illustrates the need to target both the oncogenic driver and compensatory signaling mechanisms [114,115,116,117].

Direct inhibition of KRAS G12C has also expanded treatment options for a small, molecularly defined subgroup of CRC. However, responses to KRAS G12C inhibitor monotherapy are limited by adaptive EGFR-mediated signaling. Combination approaches incorporating KRAS G12C and EGFR inhibition are therefore more biologically rational and have greater clinical relevance than single-agent treatment [118,119,120].

#### 3.2.5. CLDN18.2- and FGFR2-Directed Therapies

**Figure 5:**
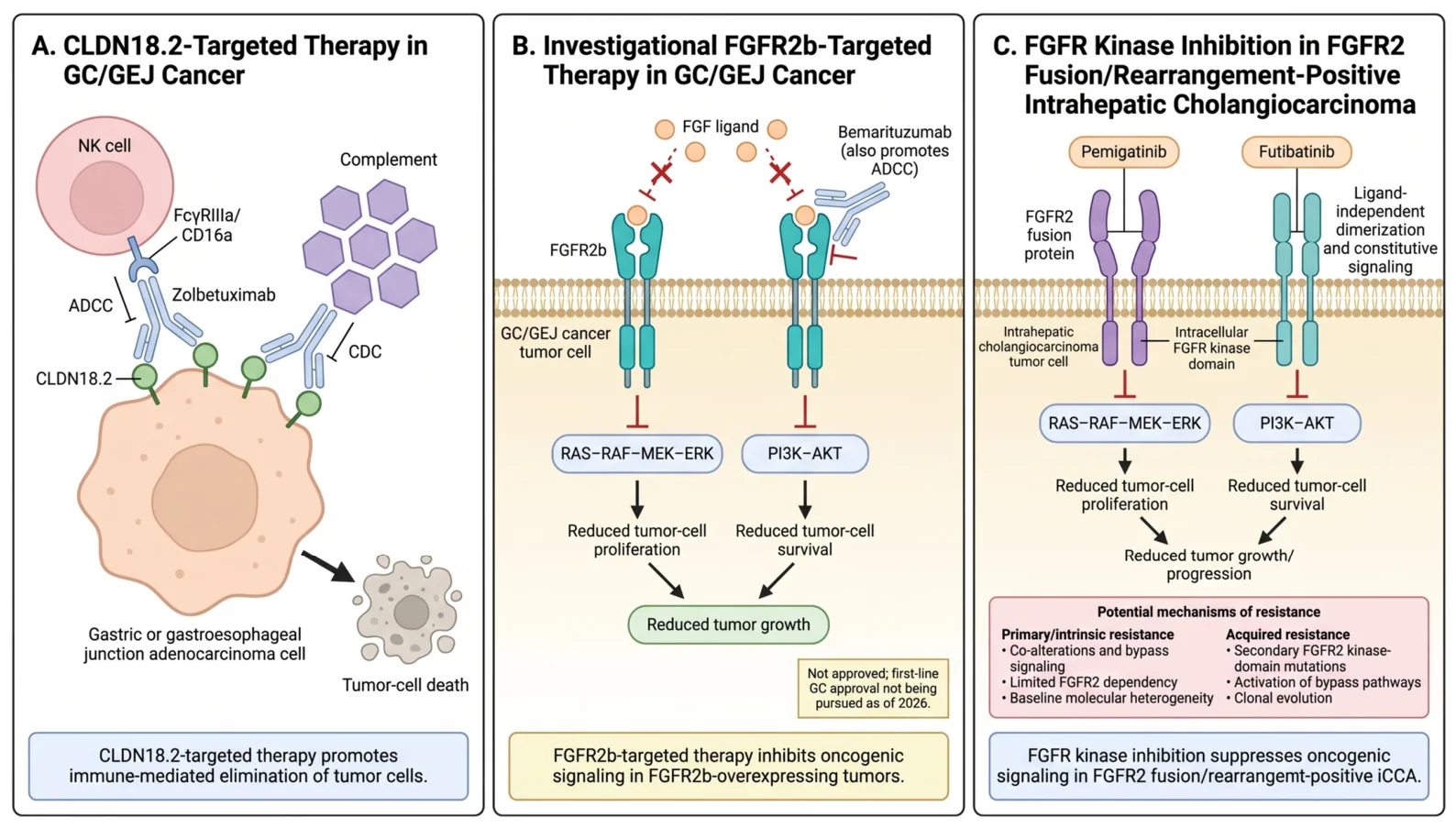
CLDN18.2- and FGFR2-directed therapeutic strategies in gastrointestinal cancers [38,39,40,41,42,43,121,126,127,128,129]. (**A**) Zolbetuximab targets CLDN18.2 and induces antibody-dependent cellular cytotoxicity (ADCC) and complement-dependent cytotoxicity (CDC) in gastric and gastroesophageal junction cancers. (**B**) Investigational bemarituzumab blocks FGFR2b signaling and may additionally promote ADCC, reducing tumor-cell proliferation, survival, and growth. (**C**) Pemigatinib and futibatinib inhibit the intracellular kinase domain of FGFR2 fusion or rearrangement proteins in intrahepatic cholangiocarcinoma, suppressing oncogenic signaling. Potential resistance mechanisms include bypass signaling, molecular heterogeneity, secondary FGFR2 mutations, and clonal evolution. Abbreviations: CLDN18.2, claudin 18.2; FGFR2b, fibroblast growth factor receptor 2b; GC, gastric cancer; GEJ, gastroesophageal junction; iCCA, intrahepatic cholangiocarcinoma; ADCC, antibody-dependent cellular cytotoxicity; CDC, complement-dependent cytotoxicity. Created in BioRender. Mertowski, S. (2026) https://BioRender.com/wgsbmpm (accessed on 12 August 2026).

CLDN18.2 and distinct FGFR2-related alterations represent separate biomarker-defined therapeutic targets in GI cancers. CLDN18.2 and the FGFR2b isoform are primarily relevant in GC and GEJ cancer, whereas *FGFR2* fusions occur mainly in selected BTCs, particularly intrahepatic cholangiocarcinoma (Figure 5). These alterations require different diagnostic approaches and are targeted by different therapeutic classes. CLDN18.2 is a tight-junction protein isoform physiologically expressed mainly in differentiated gastric mucosal cells. During malignant transformation, disruption of epithelial polarity exposes CLDN18.2 on the tumor-cell surface, making it accessible to antibody-based therapy [121,122,123,124,125]. Zolbetuximab is a CLDN18.2-directed mAb that mediates antitumor activity primarily through ADCC and CDC. Its clinical role is established in selected patients with CLDN18.2-positive, HER2-negative GC or GEJ adenocarcinoma, further refining biomarker-based stratification alongside HER2, PD-L1, and MSI/dMMR [121,126,127,128,129].

FGFR2b is an epithelial isoform of FGFR2 that may be overexpressed or amplified in a subgroup of GC and GEJ cancers. Aberrant FGFR2b signaling promotes tumor-cell proliferation and survival through downstream pathways, including RAS/RAF/MEK/ERK and PI3K/AKT. Bemarituzumab is an anti-FGFR2b mAb that blocks ligand binding and receptor activation and is being evaluated in patients with FGFR2b-positive tumors. Although clinically promising, it should currently be regarded as an emerging rather than routinely established treatment option [45,130,131,132,133,134,135].

This antibody-based strategy should be distinguished from the treatment of *FGFR2* fusion-positive BTCs. Such fusions occur predominantly in intrahepatic cholangiocarcinoma and generate constitutively active fusion proteins that drive oncogenic signaling. In this setting, small-molecule FGFR tyrosine kinase inhibitors, including pemigatinib and futibatinib, are used in selected patients with previously treated, unresectable or advanced *FGFR2* fusion- or rearrangement-positive disease [38,39,40,41,42,43,45,130,131,132,133,134,135]. The efficacy of FGFR inhibitors may be limited by primary or acquired resistance, including secondary mutations within the FGFR2 kinase domain, activation of alternative signaling pathways, and molecular heterogeneity. These limitations support the use of validated molecular testing and, where feasible, repeat molecular profiling at disease progression [136,137,138].

#### 3.2.6. IDH1-, PARP-, and Other Biomarker-Defined Therapies

**Figure 6:**
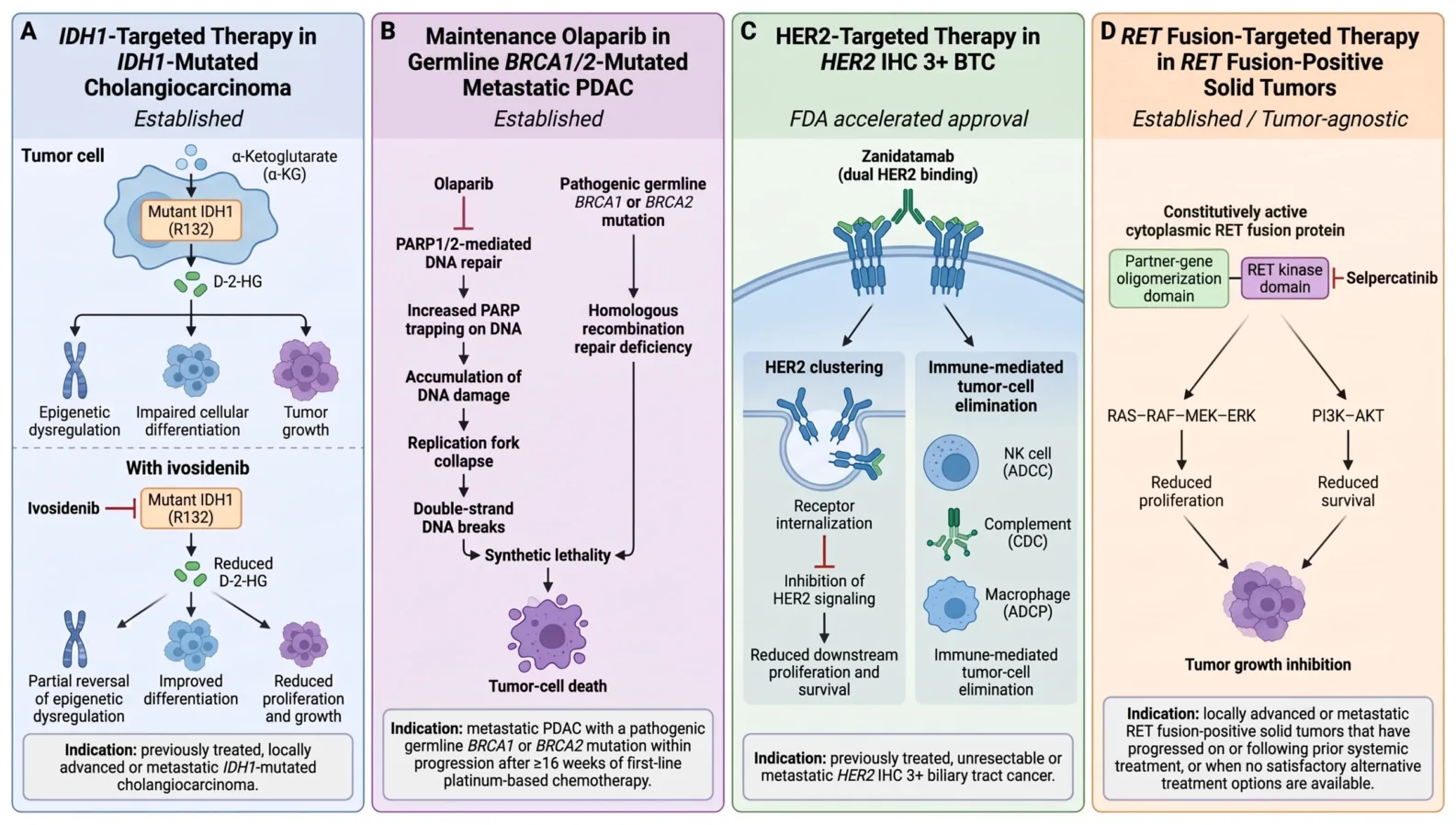
IDH1-, PARP-, HER2-, and RET-directed biomarker-defined therapies in gastrointestinal cancers. (**A**) Ivosidenib inhibits mutant IDH1, reduces D-2-hydroxyglutarate production, partially reverses epigenetic dysregulation, and limits tumor growth in previously treated, advanced IDH1-mutated cholangiocarcinoma. (**B**) Maintenance olaparib inhibits PARP-mediated DNA repair and promotes PARP trapping, leading to replication-fork collapse, double-strand DNA breaks, and synthetic lethality in metastatic PDAC with a pathogenic germline BRCA1 or BRCA2 mutation whose disease has not progressed after at least 16 weeks of first-line platinum-based chemotherapy. (**C**) Zanidatamab binds two extracellular HER2 epitopes, promotes receptor clustering and internalization, suppresses HER2 signaling, and induces immune-mediated tumor-cell elimination in previously treated, unresectable or metastatic HER2 IHC 3+ BTC. (**D**) Selpercatinib inhibits the RET kinase domain of constitutively active RET fusion proteins, suppressing RAS–RAF–MEK–ERK and PI3K–AKT signaling in locally advanced or metastatic RET fusion-positive solid tumors. Abbreviations: ADCC, antibody-dependent cellular cytotoxicity; ADCP, antibody-dependent cellular phagocytosis; BTC, biliary tract cancer; CDC, complement-dependent cytotoxicity; D-2-HG, D-2-hydroxyglutarate; HER2, human epidermal growth factor receptor 2; IDH1, isocitrate dehydrogenase 1; PARP, poly(ADP-ribose) polymerase; PDAC, pancreatic ductal adenocarcinoma; RET, rearranged during transfection [140,141,142,143,144,145,146,147,148,149]. Created in BioRender. Mertowski, S. (2026) https://BioRender.com/wgsbmpm (accessed on 12 August 2026).

Beyond the major therapeutic pathways described above, molecular profiling has identified additional actionable alterations in selected BTC and PDAC subgroups. The most clinically relevant examples include *IDH1* mutations in intrahepatic cholangiocarcinoma and defects in homologous recombination repair in PDAC. Other rare alterations, including HER2 amplification and *RET* fusions, further support the use of broad molecular profiling in advanced GI cancers (Figure 6) [139,140,141]. Mutant IDH1 acquires neomorphic enzymatic activity and converts α-ketoglutarate into the oncometabolite D-2-hydroxyglutarate. Its accumulation contributes to epigenetic dysregulation, impaired cellular differentiation, and tumor development. *IDH1* mutations occur predominantly in intrahepatic cholangiocarcinoma and provide a rationale for mutation-specific inhibition. Ivosidenib is an oral inhibitor of mutant IDH1 that reduces D-2-hydroxyglutarate production. In the phase III ClarIDHy trial, ivosidenib significantly improved progression-free survival compared with placebo in previously treated patients with advanced *IDH1*-mutated cholangiocarcinoma, establishing *IDH1* testing as an important component of molecular profiling in BTCs [141,142].

A second clinically relevant strategy involves targeting homologous recombination repair deficiency in PDAC. Pathogenic alterations in *BRCA1*, *BRCA2*, or *PALB2* impair the repair of DNA double-strand breaks and may increase sensitivity to platinum-based chemotherapy and PARP inhibition. PARP inhibitors block the repair of single-strand DNA damage and trap PARP proteins on DNA, promoting replication-associated damage and synthetic lethality in homologous recombination-deficient tumor cells [143,144,145]. The strongest evidence concerns maintenance olaparib in patients with metastatic PDAC carrying germline *BRCA1* or *BRCA2* mutations whose disease has not progressed during first-line platinum-based chemotherapy. In the phase III POLO trial, olaparib significantly prolonged progression-free survival compared with placebo, although no overall-survival advantage was demonstrated in the primary analysis [140].

The role of PARP inhibition in patients with somatic *BRCA1/2* alterations, *PALB2* mutations, or other homologous recombination repair defects is less firmly established. A phase II study of maintenance rucaparib demonstrated activity in platinum-sensitive pancreatic cancer with pathogenic germline or somatic alterations in *BRCA1*, *BRCA2*, or *PALB2*, supporting further investigation of PARP inhibitors beyond the germline *BRCA1/2* population [146]. However, these broader molecular contexts should not be considered equivalent to the established olaparib maintenance indication, and treatment decisions require careful interpretation of the specific alteration and clinical setting.

HER2 amplification or overexpression represents another actionable alteration in selected BTCs, particularly gallbladder cancer and extrahepatic cholangiocarcinoma. Zanidatamab, a bispecific antibody targeting two HER2 epitopes, has demonstrated clinically meaningful activity in previously treated, HER2-amplified, unresectable or metastatic BTC in the phase IIb HERIZON-BTC-01 study [147].

Rare *RET* fusions may also provide opportunities for tumor-agnostic treatment with selective RET inhibitors. Selpercatinib demonstrated activity across several non-lung and non-thyroid *RET* fusion-positive solid tumors in the LIBRETTO-001 basket trial, although GI-specific evidence remains limited by the rarity of these alterations and small disease-specific cohorts [148].

#### 3.2.7. Tumor-Agnostic Therapies and TRK Inhibitors

Tumor-agnostic therapy represents a shift from treatment based primarily on anatomical origin and histology toward patient selection according to a specific molecular or immunological alteration. This approach is particularly relevant in GI oncology because some actionable biomarkers occur only in small patient subgroups but may substantially expand therapeutic options when identified [9,150,151].

The best-established tumor-agnostic biomarkers in GI cancers are MSI-H/dMMR and *NTRK* gene fusions. MSI-H/dMMR tumors accumulate somatic mutations and neoantigens as a consequence of impaired DNA mismatch repair, resulting in increased immunogenicity and sensitivity to immune checkpoint blockade. Although the frequency and clinical setting of MSI-H/dMMR vary across GI malignancies, this phenotype provides a well-established basis for tumor-agnostic treatment [152,153,154].

*NTRK1*, *NTRK2*, and *NTRK3* encode the TRKA, TRKB, and TRKC proteins, respectively. Oncogenic *NTRK* fusions join the kinase domain of a TRK receptor to a partner gene, leading to ligand-independent receptor activation and persistent downstream signaling. This promotes tumor-cell proliferation, survival, migration, and oncogenic transformation. *NTRK* fusions are rare in common GI malignancies, but their detection is clinically important because they identify patients who may benefit from selective TRK inhibition [152,155,156,157,158].

Larotrectinib and entrectinib are the principal tumor-agnostic therapies used in *NTRK* fusion-positive solid tumors. Larotrectinib is a selective TRK inhibitor, whereas entrectinib also inhibits ROS1 and ALK. Both agents suppress constitutive TRK kinase activity and downstream oncogenic signaling. Their activity across multiple histological types demonstrates that, in selected molecular contexts, the oncogenic driver may be more therapeutically relevant than the anatomical site of tumor origin [155,156,157,158].

Because *NTRK* fusions and other tumor-agnostic alterations are uncommon, broad molecular profiling may be more efficient than sequential single-gene testing, particularly in advanced or refractory disease, rare histological subtypes, tumors lacking more common actionable alterations, and patients with limited standard treatment options. NGS, particularly RNA-based approaches, may facilitate fusion detection. IHC may serve as a screening method, but positive results should be confirmed using a validated molecular assay before treatment selection. The tumor-agnostic model also has important limitations. Evidence is frequently derived from basket trials that include multiple tumor types and relatively small numbers of patients with individual GI malignancies. Consequently, response estimates may not fully reflect disease-specific biology, coexisting molecular alterations, or mechanisms of resistance. Clinical implementation also depends on tissue quality, access to comprehensive molecular testing, interpretation of rare variants, and the availability of matched treatment [9,150,151,152,153,154,155,156,157,158,159,160].

### 3.3. Predictive Biomarkers and Patient Selection

Predictive biomarkers are central to treatment selection in GI oncology, but their clinical value varies according to tumor type, histological subtype, disease stage, treatment regimen, and diagnostic method. Some biomarkers directly identify an actionable molecular alteration, whereas others provide only a context-dependent estimate of treatment sensitivity. Biomarker results should therefore be interpreted within the relevant clinical indication rather than as isolated predictors of benefit [10,161,162,163,164].

MSI-H/dMMR remains the most consistently validated predictive biomarker for immune checkpoint blockade in GI cancers. Its role is particularly well established in CRC, including advanced disease, while emerging evidence supports its relevance in selected neoadjuvant settings. However, the prevalence and clinical implications of MSI-H/dMMR differ among GI malignancies, and evidence from CRC should not be transferred directly to other tumor types [55,56,57,152,153,154].

The predictive value of PD-L1 is less uniform. Its clinical relevance depends on tumor location, histology, assay, scoring system, cut-off value, treatment regimen, and line of therapy. CPS includes PD-L1 expression in tumor and selected immune cells, whereas TPS reflects the proportion of PD-L1-positive tumor cells. In GC, GEJ cancer, and ESCC, CPS is commonly used, but clinically relevant thresholds differ across indications. PD-L1 should therefore be regarded as an indication-specific biomarker rather than a universal predictor of response across GI cancers [59,60,61,62].

Other biomarkers provide a more direct link between a molecular alteration and a matched therapy. These include HER2 expression or *ERBB2* amplification, RAS WT status, *BRAF* V600E and *KRAS* G12C mutations, CLDN18.2 and FGFR2b expression, *FGFR2* fusions, *IDH1* mutations, HRR defects, and *NTRK* fusions. Their principal clinical applications, assessment methods, and limitations are summarized in Table 2.

Despite the growing number of actionable biomarkers, their assessment at a single time point may not fully capture the biological complexity of GI cancers. Spatial heterogeneity can result in substantial differences in biomarker expression within the same lesion and between primary and metastatic sites, while temporal heterogeneity may emerge through clonal evolution, treatment pressure, and disease progression. These challenges are particularly relevant for HER2, PD-L1, CLDN18.2, and FGFR2b, whose expression may vary across tumor regions and over time. As a result, treatment decisions based on a limited biopsy specimen may lead to false-negative or otherwise unrepresentative classification [74,75,76,122,123,124,125,126,127,128,129,130,131,132,133,134,135]. Previous therapy may further modify the biomarker profile by suppressing sensitive clones and promoting the expansion of resistant populations. Primary or acquired resistance may arise through secondary target alterations, activation of downstream or bypass signaling pathways, loss of target expression, or selection of pre-existing resistant subclones. Accordingly, the presence of an actionable biomarker at diagnosis does not necessarily ensure sustained treatment sensitivity throughout the disease course [163].

When clinically feasible, reassessment at progression—through repeat tissue biopsy or updated molecular profiling—may help identify biomarker discordance, acquired resistance, or newly actionable alterations. However, repeated sampling is not always possible because of limited tumor accessibility, insufficient tissue quality, procedure-related risk, or restricted access to validated diagnostic assays. These limitations highlight the need to interpret biomarker results as dynamic and context-dependent rather than as fixed characteristics of the tumor [164,165].

### 3.4. Integration of Biological and Targeted Therapies with Surgery

**Figure 7:**
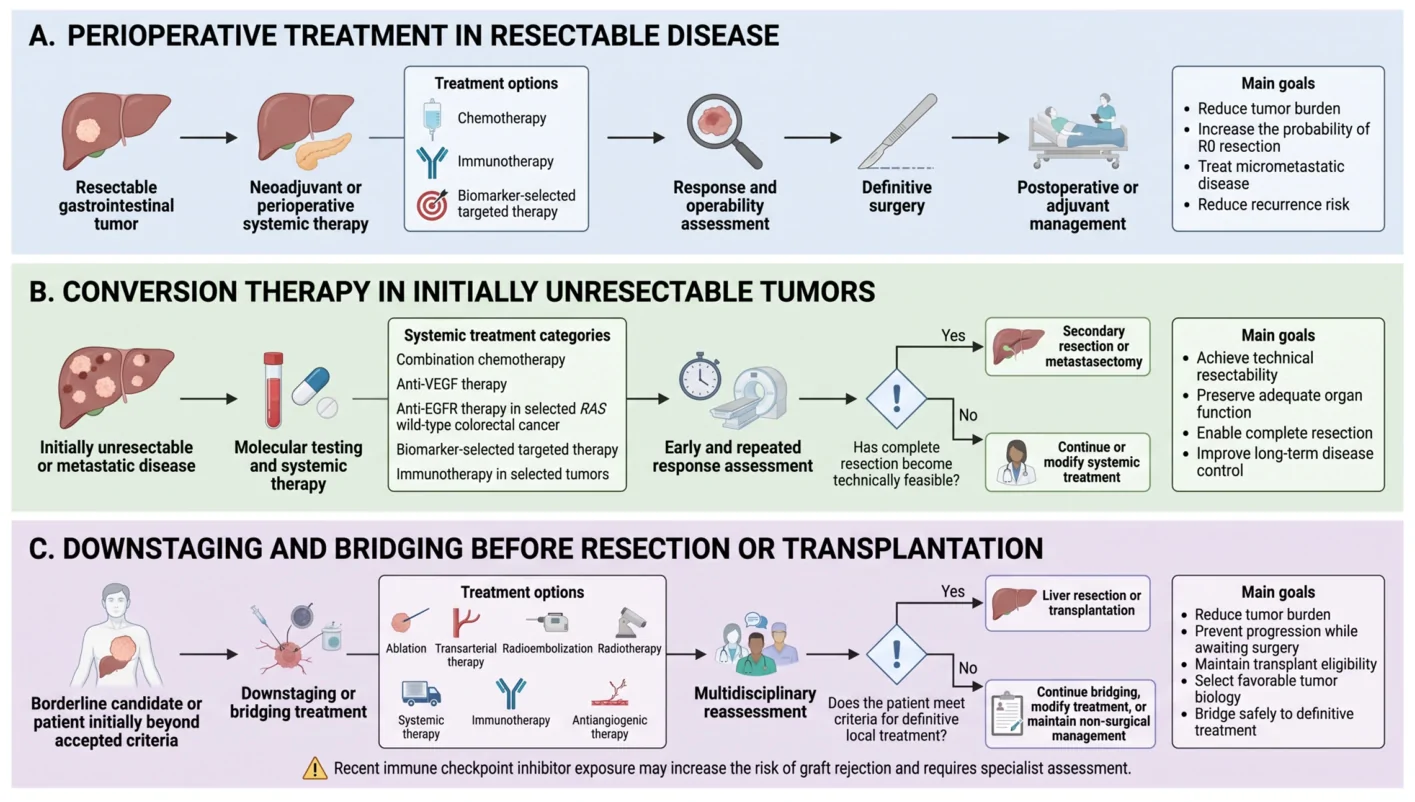
Three main models of integration of systemic therapy with surgery in GI cancers [60,166,167,168,169,170]. Biological and targeted therapies may be used as perioperative treatment in resectable disease, as conversion therapy in initially unresectable tumors, or as downstaging and bridging treatment before resection or transplantation. Surgical decisions require repeated assessment of treatment response, anatomical resectability, biomarker context, organ function, treatment-related toxicity, and overall clinical status by a multidisciplinary team. Created in BioRender. Mertowski, S. (2026) https://BioRender.com/wgsbmpm (accessed on 12 August 2026). In resectable esophageal, GC, and GEJ cancers, systemic therapy is increasingly integrated before and after surgery to reduce tumor burden, address micrometastatic disease, and decrease recurrence risk. Adjuvant nivolumab is established for selected patients with residual pathological disease after neoadjuvant chemoradiotherapy and R0 resection of esophageal or GEJ cancer. In GC and GEJ adenocarcinoma, perioperative chemotherapy remains central, while immunotherapy-containing regimens are entering selected perioperative settings. However, treatment response should not routinely justify reducing the oncological extent of surgery or omitting resection outside validated protocols [19,20,27,28,171,172,173]. CheckMate 577 demonstrated longer disease-free survival with adjuvant nivolumab in patients with residual disease after neoadjuvant chemoradiotherapy and complete resection [171,172].

The integration of biological and targeted therapies with surgery has become an increasingly important component of multimodal treatment in GI oncology. These therapies may support surgical management in three principal ways: as perioperative treatment in resectable disease, as conversion therapy in initially unresectable tumors, or as downstaging and bridging treatment before resection or transplantation. These three models are summarized in Figure 7. In each setting, the aim of systemic treatment extends beyond tumor shrinkage and includes eradication of micrometastatic disease, assessment of tumor biology, improvement of the probability of complete resection, and selection of patients most likely to benefit from an invasive procedure.

Surgery after preoperative immunotherapy is generally feasible, but inflammation, edema, fibrosis, adhesions, and altered tissue planes may increase operative complexity in individual patients. Preoperative assessment should therefore consider unresolved irAEs, corticosteroid exposure, cardiopulmonary and endocrine function, liver function, and the timing of the last treatment dose [173,174].

Neoadjuvant checkpoint blockade has produced particularly strong responses in localized dMMR/MSI-H CRC. In dMMR rectal cancer, clinical complete responses after PD-1 blockade have created the possibility of organ-preserving management under intensive clinical, endoscopic, and radiological surveillance. Nevertheless, the evidence is derived from selected cohorts, and longer follow-up is required before surgery can be routinely omitted [175].

In dMMR colon cancer, short-course neoadjuvant nivolumab plus ipilimumab has produced high major and complete pathological response rates without generally preventing timely surgery. However, colectomy remains the standard definitive treatment, and pathological sensitivity should not yet be interpreted as justification for routine nonoperative management [176].

In metastatic CRC, conversion therapy may enable secondary resection of initially unresectable liver- or lung-limited metastases. Treatment selection should consider RAS and BRAF status, primary tumor sidedness, metastatic distribution, and the need for rapid and deep tumor shrinkage. Anti-EGFR-based combinations may be particularly useful in selected RAS WT, left-sided tumors, whereas chemotherapy combined with anti-VEGF therapy remains an important alternative across broader molecular subgroups [25,34,36,177,178,179].

Radiological response alone should not determine surgical eligibility. Disappearing lesions may still contain viable tumors, whereas complete radiological resolution is not required for technically successful resection. Early and repeated multidisciplinary reassessment is therefore essential, and surgery should be considered once complete resection becomes feasible while preserving adequate organ function, rather than after prolonged treatment until maximal response [180,181].

Anti-VEGF treatment requires specific perioperative planning because VEGF inhibition may impair wound healing and increase the risk of bleeding, dehiscence, thrombosis, and anastomotic complications. The interval between treatment and surgery should be individualized according to the agent, extent of surgery, comorbidities, and wound-healing risk, and treatment should be resumed only after adequate postoperative recovery [182,183]. Increased wound-healing complications have been reported when major surgery was performed during bevacizumab treatment.

In HCC, downstaging and bridging strategies may enable resection or liver transplantation or maintain disease control while a patient awaits definitive treatment. Locoregional therapies remain the most established approaches, whereas immunotherapy and antiangiogenic combinations are emerging options in selected patients. Surgical or transplant eligibility should be based not only on radiological response but also on liver function, portal hypertension, AFP dynamics, extrahepatic spread, and the stability of tumor biology. ICI exposure before transplantation requires particular caution because persistent immune activation may increase graft-rejection risk [37,38,184,185].

In BTCs and PDAC, secondary resection after systemic or biomarker-directed therapy remains uncommon. Induction therapy may occasionally permit resection in locally advanced PDAC, while responses to FGFR-, IDH1-, HER2-, or immunotherapy-based treatment may prompt reassessment in selected BTCs. However, evidence is derived mainly from retrospective studies, small cohorts, and case reports. Secondary resection should therefore not be considered routine and requires sustained disease control, technical feasibility, acceptable operative risk, and absence of uncontrolled metastatic progression [31,32,33,182,183,186,187]. The principal clinical scenarios in which biological and targeted therapies may be integrated with surgery are summarized in Table 3.

### 3.5. General Regulatory Status and Presence on Polish Reimbursement Lists

To assess treatment availability, three related but not identical aspects were distinguished: the presence of a biological therapeutic target, registration of the therapy for a specific indication, and reimbursement of a given indication or treatment regimen in the Polish public health system. The identification of a predictive biomarker, such as MSI-H/dMMR, PD-L1, HER2, alterations in the RAS, BRAF, KRAS G12C, NTRK, FGFR2, IDH1, BRCA1/2 genes, or CLDN18.2 expression, may indicate potential biological eligibility for molecularly matched therapy. However, the presence of a biomarker alone does not confirm registration or reimbursement of treatment for a specific cancer type, disease stage, line of therapy, or regimen [194,195,196,197,198,199,200,201,202,203,204,205,206,207,208]. Therefore, the registration and reimbursement status was assessed at the level of individual indications and treatment regimens, not solely the medicinal product. Table 4 presents the indication, required biomarker or diagnostic criterion, disease stage, clinical context, relevant prior therapy, FDA and EMA regulatory indications, and reimbursement status in Poland as of 1 January 2026 [209,210,211,212,213,214,215,216,217,218,219,220,221,222,223,224,225,226,227,228,229,230,231,232,233,234,235,236,237,238,239,240,241,242,243,244,245,246,247,248,249,250,251,252,253,254,255,256,257,258,259,260,261,262,263,264,265,266,267,268,269,270,271,272,273,274]. The FDA and EMA regulatory status is presented separately due to potential differences in indications, including biomarker thresholds, histological subtype, disease stage, line of treatment, requirements for prior therapy, and permissible combination regimens. This was particularly true for immunotherapy in upper gastrointestinal cancers, for which the required PD-L1 expression thresholds may differ between the United States and the European Union.

In the case of the assessed Polish reimbursement status, the official announcement effective January 1, 2026, as well as the relevant drug programs and chemotherapy catalog entries [211], programs B.58, B.4, B.5, B.85, and B.144, were considered. As mentioned above, the presence of a product in reimbursement documents was not automatically considered reimbursement for all of its registered indications. If a specific cancer or treatment regimen was not listed, it was classified as “unidentified as of the cutoff date,” even if the product was reimbursed for another indication. For example, as of the cutoff date, no reimbursement was identified for pembrolizumab in combination with gemcitabine and cisplatin in biliary tract cancer, the combination of nivolumab with ipilimumab, or durvalumab with tremelimumab in hepatocellular carcinoma. Sotorasib, ivosidenib, and dostarlimab were included in reimbursement documents for other cancers, but not for the analyzed gastrointestinal indications. This confirms the need to interpret reimbursement based on the specific cancer type, biomarker, regimen, and clinical context. At the same time, reimbursed, indication-related regimens were identified, including pembrolizumab, nivolumab, tislelizumab, trastuzumab, trastuzumab deruxtecan, and ramucirumab for upper gastrointestinal cancers; pembrolizumab and nivolumab with ipilimumab for MSI-H/dMMR colorectal cancer; atezolizumab with bevacizumab for hepatocellular carcinoma; durvalumab with gemcitabine and cisplatin for biliary tract cancer; olaparib for pancreatic cancer with a germline BRCA1/2 mutation; and larotrectinib and entrectinib for solid tumors with an NTRK fusion.

The cutoff date was strictly applied. Regulatory opinions, indication extensions, and reimbursement decisions issued after 1 January 2026 were not included in the classification. The analysis did not include comprehensive criteria for drug programs, local availability of services, hospital contracting, waiting times, or individual patient eligibility. Table 4 should therefore be interpreted as an indication- and time-dependent summary of formal FDA and EMA registration status and reimbursement status in Poland, rather than a comprehensive assessment of the actual clinical availability of the treatment.

### 3.6. Emerging Directions in Precision Oncology

Precision oncology in GI cancers is progressing from single-biomarker testing toward multidimensional and longitudinal characterization of tumor biology. Genomic profiling remains fundamental for detecting mutations, amplifications, copy-number alterations, and gene fusions, but genomic information alone does not fully reflect pathway activity, cellular phenotype, immune interactions, or treatment-induced adaptation. Integration of genomic data with transcriptomics, proteomics, metabolomics, epigenomics, digital pathology, imaging, and clinical variables may therefore improve molecular classification, biomarker discovery, and prediction of treatment response [275,276,277].

Transcriptomic analysis provides information on gene-expression programs and pathway activation, whereas proteomic and phosphoproteomic approaches assess functional protein abundance and signaling activity. Metabolomic profiling may identify metabolic dependencies associated with tumor progression, immune suppression, and therapeutic resistance. When these data are analyzed jointly, they can distinguish biologically distinct tumors that share the same genomic alteration but differ in downstream signaling or treatment sensitivity. However, multiomic integration remains limited by differences in analytical platforms, sample processing, computational pipelines, data completeness, and the difficulty of validating complex molecular signatures in independent cohorts [275,276].

Single-cell sequencing provides an additional level of resolution by separating malignant, immune, stromal, endothelial, and other cellular populations that are averaged together in bulk-tissue analyses. Single-cell studies in GC and CRC have revealed extensive diversity of malignant-cell states, cancer-associated fibroblasts, myeloid populations, and lymphocyte subsets, illustrating how cellular composition and functional state may influence progression and response to treatment [278,279]. Nevertheless, dissociation-based single-cell methods partially remove cells from their original anatomical context and may underrepresent fragile or poorly recovered cell populations.

Spatial transcriptomic and proteomic approaches address this limitation by preserving the localization of molecular signals within intact tissue. These methods can identify immune-excluded regions, stromal barriers, invasive fronts, tertiary lymphoid structures, resistant cellular niches, and spatially restricted communication between tumor and immune cells. Their application may improve understanding of why tumors with apparently similar genomic profiles show different responses to ICIs or targeted therapies. At present, however, spatial profiling remains primarily a translational research tool because of its cost, tissue requirements, platform variability, computational complexity, and lack of clinically validated decision thresholds [277,279]. Spatial profiling technologies increasingly enable characterization of tumor-cell architecture and interactions that are not detectable using bulk analyses alone.

Liquid biopsy offers a complementary approach by enabling minimally invasive and repeated molecular assessment. ctDNA can be used to identify tumor-derived alterations when tissue is unavailable or insufficient, monitor molecular response during treatment, detect emerging resistant clones, and assess molecular residual disease after curative-intent surgery. Because ctDNA may originate from several tumor sites simultaneously, it may also provide a broader representation of systemic disease than a single tissue biopsy. However, its sensitivity depends on tumor burden, anatomical location, vascularity, assay design, timing of blood collection, and the biological rate of DNA shedding [280,281,282].

The strongest evidence for postoperative ctDNA assessment in GI oncology currently concerns CRC. In the DYNAMIC trial, ctDNA-guided management of stage II colon cancer reduced the use of adjuvant chemotherapy without compromising recurrence-free survival [283]. In the CIRCULATE-Japan GALAXY study, postoperative ctDNA positivity was strongly associated with recurrence and inferior survival, while sustained ctDNA clearance during adjuvant treatment was associated with more favorable outcomes [218]. These findings support the prognostic and potential treatment-guiding value of molecular residual disease assessment, although the optimal assay, sampling schedule, and management of ctDNA-positive patients remain under investigation.

Emerging evidence also supports longitudinal ctDNA monitoring in other GI cancers. In resectable GC and GEJ cancer, ctDNA detected after preoperative therapy or surgery has been associated with pathological response, recurrence risk, and survival. Prospective observations suggest that persistent or re-emerging ctDNA may identify molecular non-response or residual disease before conventional clinical progression [284,285]. Nevertheless, evidence outside CRC remains less mature, and ctDNA-guided treatment changes have not yet been established as routine standards for most GC, PDAC, BTC, or HCC settings.

Serial molecular profiling may also support adaptive treatment strategies. Repeated tissue or liquid-biopsy analysis can reveal loss of the original therapeutic target, emergence of secondary kinase-domain mutations, activation of bypass pathways, expansion of resistant subclones, or acquisition of a new actionable alteration. In selected settings, these findings may support treatment escalation, de-escalation, switching to another targeted agent, or molecularly guided rechallenge. For example, serial ctDNA analysis may identify the emergence or disappearance of resistant RAS or EGFR-pathway clones during anti-EGFR treatment in CRC. However, detecting molecular evolution does not automatically establish that changing treatment on this basis improves survival.

The implementation of adaptive precision oncology is also limited by analytical and logistical challenges. These include incomplete assay standardization, variable sensitivity and specificity, clonal hematopoiesis, low ctDNA shedding, uncertain thresholds for intervention, insufficient evidence for many detected variants, high costs, restricted reimbursement, and unequal access to specialized laboratories and bioinformatic infrastructure. Prospective trials must therefore demonstrate not only analytical validity but also clinical utility—that is, whether a molecularly triggered treatment change produces better outcomes than standard radiological and clinical monitoring. The principal emerging technologies, their potential clinical applications, and current limitations are summarized in Table 5.

## 4. Discussion

The therapeutic landscape of GI cancers is shifting from a predominantly organ- and histology-based model toward an increasingly biomarker-informed and context-dependent approach. This transition does not replace the importance of anatomical site, histological subtype, disease stage, or resectability. Instead, molecular and immunological characteristics have become additional determinants of treatment selection. Consequently, tumors arising in the same organ may require different therapeutic strategies, whereas malignancies of different anatomical origin may become eligible for the same treatment when they share a clinically actionable alteration. MSI-H/dMMR tumors and cancers harboring oncogenic *NTRK* fusions provide the clearest examples of this tumor-agnostic principle [9,287,288,289,290].

The reviewed evidence identifies three broad models of precision treatment. The first includes therapies requiring direct demonstration of a molecular target or predictive alteration, such as HER2-, EGFR-, CLDN18.2-, FGFR2-, IDH1-, and HRR-directed strategies. The second includes agents targeting biological processes shared across multiple tumor types, exemplified by antiangiogenic treatment. The third is immunotherapy, whose efficacy depends on the interaction between tumor cells, genomic instability, and the immune microenvironment. Within this group, MSI-H/dMMR remains the most consistently validated predictive biomarker, whereas PD-L1 expression is more dependent on tumor type, scoring method, cut-off, treatment regimen, and line of therapy [11,165,291,292,293,294,295,296,297].

A central finding of this review is that biomarker relevance cannot be separated from disease-specific context. Molecular alterations such as RAS and *BRAF* mutations or *FGFR2* and *NTRK* fusions are relatively well defined, whereas HER2, PD-L1, CLDN18.2, and FGFR2b depend more strongly on expression thresholds, assay methodology, tissue quality, and sampling location. Spatial and temporal heterogeneity may therefore produce discordance within the same tumor, between primary and metastatic lesions, or between samples obtained before and after treatment. Moreover, primary and acquired resistance may develop through secondary target alterations, downstream pathway reactivation, bypass signaling, loss of target expression, or expansion of resistant subclones. Thus, detection of an actionable biomarker at diagnosis does not guarantee durable treatment sensitivity.

The growing integration of biological and targeted therapies with surgery further illustrates the need for individualized decision-making. Surgery remains the principal curative modality for most localized GI cancers, but its timing and role are increasingly modified by perioperative, conversion, and downstaging strategies. In resectable disease, systemic therapy may increase pathological response and reduce recurrence risk. In initially unresectable metastatic CRC, treatment may enable secondary resection of liver- or lung-limited disease. In selected dMMR rectal cancers, complete clinical response may support organ-preserving management, whereas in HCC, downstaging and bridging may facilitate resection or liver transplantation. Nevertheless, radiological or clinical response should not automatically be equated with complete pathological eradication, technical resectability, or cure, and repeated multidisciplinary assessment remains essential [289,290,291,292].

The strength of evidence supporting these strategies varies considerably. Several indications are based on randomized trials and have become established components of care, whereas newer approaches may rely on single-arm studies, basket trials, retrospective cohorts, or individual reports. Basket trials are particularly important for rare molecular alterations, but the small number of patients with individual GI cancers may limit disease-specific interpretation. Regulatory authorization across multiple solid tumors should therefore not be considered equivalent evidence of benefit in every GI malignancy.

Another important issue is the distinction between biological actionability, regulatory authorization, and presence on reimbursement lists. Identification of a therapeutic target does not confirm that a corresponding drug has been authorized, while FDA or EMA authorization does not automatically imply public funding. Similarly, the presence of a product on a Polish reimbursement list does not establish reimbursement for every indication, treatment line, regimen, or biomarker-defined subgroup. The regulatory analysis presented in this review should therefore be interpreted as a time-specific overview of formal status rather than a measure of complete clinical access [15,16,17].

Access to molecular diagnostics is equally important. Modern GI oncology increasingly requires assessment of MSI/dMMR, PD-L1, HER2, RAS, *BRAF*, *NTRK*, CLDN18.2, FGFR2b, *FGFR2*, *IDH1*, and selected HRR alterations. Inadequate tissue, delayed testing, lack of validated assays, and limited access to NGS may prevent patients from being identified for matched therapy even when a drug is formally authorized. These challenges also affect assay sequencing, turnaround time, tissue preservation, and the cost-effectiveness of broad molecular profiling [292,293,294,295,296].

Emerging multiomic, single-cell, spatial, and liquid-biopsy approaches may improve characterization of tumor heterogeneity and molecular evolution. Multiomic integration can connect genomic alterations with transcriptional programs, protein activity, metabolism, and immune composition. Single-cell and spatial methods may identify resistant cellular niches, while serial ctDNA analysis may support molecular residual-disease assessment, earlier detection of recurrence, monitoring of response, and identification of emerging resistant clones. However, routine adaptive treatment based on these technologies remains limited by incomplete standardization, uncertain intervention thresholds, variable sensitivity, cost, and insufficient prospective evidence that molecularly triggered treatment changes improve patient outcomes.

Assessment of clinical value should also extend beyond regulatory approval and response rates. New therapies differ in their effects on progression-free survival, overall survival, toxicity, quality of life, and treatment burden. This is particularly relevant for costly agents directed at small molecular subgroups. Structured frameworks such as the ESMO Magnitude of Clinical Benefit Scale may support comparison and prioritization, but they do not replace individualized clinical judgment or national health-technology assessment [297,298,299,300,301].

This review has several limitations. It is narrative rather than systematic and does not include a formal meta-analysis, quantitative comparison of treatment effects, or standardized risk-of-bias assessment. The included evidence is heterogeneous with respect to study design, cancer type, disease stage, treatment line, sample size, and follow-up. Regulatory authorization and presence on Polish reimbursement lists were assessed at a predefined time point and were not analyzed at the level of detailed indication-specific reimbursement criteria or individual patient access. In addition, rapidly evolving areas, including perioperative immunotherapy, ctDNA-guided treatment, spatial profiling, bispecific antibodies, ADCs, and cellular therapies, may be affected by new evidence or regulatory changes emerging after completion of the literature assessment.

Despite these limitations, this review integrates biological mechanisms, predictive biomarkers, clinical treatment settings, surgical implications, regulatory status, and reimbursement-list presence within a single framework. This approach reflects the practical complexity of precision oncology, in which therapeutic decision-making requires simultaneous consideration of molecular eligibility, strength of evidence, disease stage, resectability, patient condition, toxicity, diagnostic feasibility, and formal treatment status within the healthcare system.

## 5. Conclusions

Biological and targeted therapies have substantially reshaped the therapeutic landscape of GI cancers, particularly in molecularly defined subgroups. ICIs, HER2-directed agents, antiangiogenic therapies, anti-EGFR antibodies, CLDN18.2- and FGFR-directed strategies, and tumor-agnostic treatments have shifted therapeutic selection from a purely anatomical and histological model toward an integrated biomarker-driven approach. This transition is particularly evident in MSI-H/dMMR tumors, HER2-positive GC/GEJ cancers, RAS WT metastatic CRC, CLDN18.2-positive GC, and molecularly selected BTCs.

Despite these advances, surgery remains the foundation of curative-intent treatment in localized GI cancers. Biological and targeted therapies increasingly complement surgical management by modifying its timing and clinical context through neoadjuvant, perioperative, adjuvant, conversion, and downstaging strategies. Optimal treatment planning therefore requires multidisciplinary integration of tumor site, stage, resectability, molecular profile, patient condition, expected benefit, toxicity, and treatment availability.

Future progress will depend on broader access to validated molecular diagnostics, repeated assessment of tumor evolution, and integration of tissue profiling with ctDNA, multiomic, single-cell, and spatial approaches. Equally important will be the reduction in discrepancies between regulatory authorization, reimbursement-list status, and practical clinical implementation. The further development of precision oncology in GI cancers should therefore combine therapeutic innovation with robust biomarker validation, prospective evidence of clinical benefit, cost-effectiveness, and equitable access to testing and treatment.

## Figures and Tables

**Table 1:** Clinicopathological characteristics, molecular stratification, and therapeutic context of major gastrointestinal cancers.

Cancer Type	Predominant Histology	Principal Biological and Clinical Characteristics	Role of Surgery and Multimodal Treatment	Key Therapeutic Biomarkers and Molecular Alterations	Molecular Stratification in Current Practice	References
EC	Squamous cell carcinoma and adenocarcinoma	Comprises two biologically distinct histological subtypes with different epidemiology, risk factors, molecular profiles, and sensitivity to systemic treatment	Surgery is an important component of curative-intent treatment in resectable disease and is commonly integrated with neoadjuvant chemoradiotherapy or perioperative chemotherapy; adjuvant immunotherapy may be used in selected settings	PD-L1, MSI-H/dMMR; HER2 in selected distal esophageal and gastroesophageal junction adenocarcinomas	Moderate and strongly dependent on histological subtype, disease stage, and treatment setting	[19,23,27,28]
GC/GEJ cancer	Predominantly adenocarcinoma	Characterized by marked intertumoral and intratumoral heterogeneity and by the coexistence of partially overlapping biomarker-defined subgroups	Surgery remains the mainstay of curative-intent treatment in resectable disease and is usually combined with perioperative chemotherapy; immunotherapy and biomarker-directed treatment are increasingly used in advanced disease	HER2, PD-L1 CPS, MSI-H/dMMR, CLDN18.2, FGFR2b; EBV-associated subtype in selected molecular classifications	Advanced and rapidly expanding, with parallel testing of several biomarkers increasingly required	[18,19,20,21,22,23,29,30]
CRC	Predominantly adenocarcinoma	One of the best-characterized molecularly heterogeneous GI cancers, with clinically relevant differences related to primary tumor sidedness, genomic profile, and metastatic pattern	Surgery is the mainstay of treatment for localized disease; resection of selected liver or lung metastases may be feasible. Adjuvant therapy, neoadjuvant treatment in rectal cancer, and conversion therapy are used in selected clinical settings	*KRAS*, *NRAS*, *BRAF*, MSI-H/dMMR, HER2 alterations, *KRAS* G12C, *NTRK* fusions, and *PIK3CA* alterations in selected contexts	Highly advanced and routinely incorporated into systemic treatment selection	[25,26,34,35,36,37]
PDAC	Ductal adenocarcinoma	Characterized by aggressive biology, early metastatic spread, a dense stromal microenvironment, and a relatively low frequency of clinically actionable alterations	Surgical resection is the only potentially curative treatment but is feasible in a minority of patients; adjuvant chemotherapy and induction or neoadjuvant treatment are used in resectable or borderline resectable disease	*KRAS* mutations in most tumors; MSI-H/dMMR, *BRCA1*, *BRCA2*, *PALB2*, *NTRK* fusions, HER2 alterations, and other homologous recombination repair defects in small subgroups	Limited to selected molecular subgroups	[31,32,33]
HCC	Hepatocellular carcinoma	Frequently develops in chronically diseased or cirrhotic liver and is characterized by substantial molecular heterogeneity and complex interactions with the hepatic immune microenvironment	Resection, transplantation, and local ablation may provide curative treatment in selected patients with early-stage disease; locoregional and systemic therapies are used in intermediate and advanced stages	AFP in selected clinical contexts; *TERT* promoter, *CTNNB1*, *TP53*, and other alterations mainly of biological or prognostic relevance; GPC3 under investigation; no routinely established predictive biomarker for most systemic regimens	Limited for predictive treatment selection; therapeutic decisions remain driven mainly by tumor stage and liver function	[38,39]
BTCs	Intrahepatic and extrahepatic cholangiocarcinoma and gallbladder carcinoma	Represent a heterogeneous group with substantial anatomical, histological, and molecular differences between subtypes	Complete surgical resection remains the only potentially curative treatment but is available to a limited proportion of patients; chemotherapy combined with immunotherapy is used in advanced disease, followed by molecularly targeted therapy in selected subgroups	*FGFR2* fusions and *IDH1* mutations, particularly in intrahepatic cholangiocarcinoma; HER2 alterations, *BRAF* V600E, MSI-H/dMMR, and *NTRK* fusions in selected tumors	Increasingly advanced, particularly in intrahepatic cholangiocarcinoma	[24,40,41,42,43]

Abbreviations: AFP, alpha-fetoprotein; BTCs, biliary tract cancers; CLDN18.2, claudin 18.2; CPS, combined positive score; CRC, colorectal and rectal cancer; dMMR, deficient mismatch repair; EBV, Epstein–Barr virus; EC, esophageal cancer; *FGFR2*, fibroblast growth factor receptor 2; FGFR2b, fibroblast growth factor receptor 2b; GC, gastric cancer; GEJ, gastroesophageal junction; GI, gastrointestinal; GPC3, glypican-3; HCC, hepatocellular carcinoma; HER2, human epidermal growth factor receptor 2; MSI-H, microsatellite instability-high; NTRK, neurotrophic tyrosine receptor kinase; PDAC, pancreatic ductal adenocarcinoma; PD-L1, programmed death-ligand 1.

**Table 2:** Principal predictive biomarkers used for patient selection in GI cancers.

Biomarker	Principal Cancer Context	Preferred Assessment	Matched Therapeutic Strategy	Clinical Interpretation	Main Limitations	References
MSI-H/dMMR	CRC; selected GC/GEJ, BTC, PDAC, and other GI cancers	IHC for MMR proteins and/or validated PCR- or NGS-based MSI testing	ICIs	Strongest validated biomarker for checkpoint blockade; established in advanced CRC and increasingly relevant in selected neoadjuvant settings	Low prevalence in several GI cancers; possible discordance between testing methods; evidence differs by tumor type and disease setting	[55,56,57,152,153,154]
PD-L1	GC/GEJ cancer, ESCC, and selected upper-GI cancers	Validated IHC assay; most commonly CPS, with TPS used in selected settings	PD-1/PD-L1 blockade within indication-specific regimens	Context-dependent biomarker whose relevance varies by cancer type, assay, cut-off, regimen, and treatment line	Spatial and temporal heterogeneity; assay variability; different CPS or TPS thresholds; limited value as a universal biomarker	[59,60,61,62]
HER2/*ERBB2*	GC/GEJ cancer; selected BTC and CRC	IHC followed by ISH in equivocal cases; NGS in selected settings	Trastuzumab, trastuzumab deruxtecan, zanidatamab, and other HER2-directed agents	Established in selected GC/GEJ settings and increasingly relevant in molecularly defined BTC and CRC subgroups	Intratumoral heterogeneity; differences in scoring criteria; temporal changes in expression; discordance between primary and metastatic sites	[69,73,74,75,76,77,78,79,80,81,82,83,84,85,86]
RAS WT	Metastatic CRC	Validated testing of *KRAS* and *NRAS* using PCR or NGS	Cetuximab or panitumumab	Absence of activating RAS mutations is required for anti-EGFR treatment; benefit is greatest in selected left-sided tumors	Primary and acquired resistance; tumor sidedness; emergence of resistant subclones	[99,100,101,102,103,104,105,106,107,108,109]
*BRAF* V600E	Metastatic CRC; selected BTC	PCR or NGS	BRAF inhibition combined with EGFR blockade in CRC; indication-specific strategies in other cancers	Identifies an aggressive molecular subgroup and guides pathway-combination therapy	Limited efficacy of BRAF inhibitor monotherapy in CRC because of feedback EGFR activation	[35,36,103,106]
*KRAS* G12C	Selected metastatic CRC	PCR or NGS	KRAS G12C inhibitor combined with EGFR blockade	Defines a small subgroup eligible for mutation-specific treatment	Adaptive EGFR signaling; acquired resistance; low prevalence	[103]
CLDN18.2	HER2-negative GC/GEJ adenocarcinoma	Validated IHC assay	Zolbetuximab with chemotherapy	Established expression-based biomarker in selected advanced GC/GEJ cancer	Intratumoral heterogeneity; assay- and threshold-dependent classification; sampling limitations	[121,122,123,124,125,126,127,128,129]
FGFR2b	Selected GC/GEJ cancer	IHC-based assessment of FGFR2b expression	Bemarituzumab and other emerging FGFR2b-directed approaches	Promising expression-based biomarker under clinical development	Lack of universal scoring criteria; heterogeneous expression; not yet a routine standard	[45,130,131,132,133,134,135,162]
*FGFR2* fusion or rearrangement	Predominantly intrahepatic cholangiocarcinoma	DNA- or preferably RNA-based NGS; validated fusion assay	Pemigatinib or futibatinib	Established molecular target in selected previously treated advanced disease	Rare alteration; secondary kinase-domain mutations; molecular heterogeneity and acquired resistance	[40,41,42,43]
*IDH1* mutation	Intrahepatic cholangiocarcinoma	Validated PCR or NGS	Ivosidenib	Established target in selected previously treated advanced disease	Restricted mainly to a molecular subgroup of iCCA; co-alterations and acquired resistance may affect response	[141,142]
Germline *BRCA1/2* mutation	Platinum-sensitive metastatic PDAC	Germline testing, with tumor profiling where appropriate	Maintenance olaparib	Established biomarker for maintenance PARP inhibition after disease control with platinum-based chemotherapy	Limited to a defined clinical setting; benefit should not be generalized to all HRR alterations	[139]
Somatic *BRCA1/2*, *PALB2*, and other HRR alterations	Selected PDAC	Tumor NGS with germline confirmation when indicated	PARP inhibition or platinum-based strategies in selected or investigational settings	Potentially actionable, but less firmly established than germline *BRCA1/2*	Heterogeneous biological relevance; variable treatment sensitivity; insufficient evidence for several individual genes	[146]
*NTRK* fusion	Rare molecular subgroups across GI cancers	RNA-based NGS preferred; IHC may be used for screening	Larotrectinib or entrectinib	Tumor-agnostic biomarker with high therapeutic relevance when present	Very low prevalence; false-positive screening results; evidence derived mainly from basket trials	[150,151,152,153,154,155,156,157,158]

Abbreviations: CPS, combined positive score; HRR, homologous recombination repair; ICI, immune checkpoint inhibitor; MMR, mismatch repair; NGS, next-generation sequencing; PCR, polymerase chain reaction; TPS, tumor proportion score; WT, wild-type.

**Table 3:** Main clinical models of integrating biological and targeted therapies with surgery in GI cancers.

Clinical Context	Main Strategy	Surgical Objective	Key Selection Factors	Evidence Status	References
Resectable esophageal and GC/GEJ cancers	Perioperative or adjuvant systemic therapy, including immunotherapy in selected settings	Improve pathological response and reduce recurrence risk	Stage, histology, treatment response, residual pathological disease, fitness for surgery	Established in selected esophageal/GEJ settings; perioperative immunotherapy in GC/GEJ is rapidly evolving	[173,174,188]
Localized dMMR/MSI-H CRC	Neoadjuvant checkpoint blockade	Facilitate surgery or support organ preservation in selected rectal cancer	Confirmed dMMR/MSI-H, reliable response assessment, capacity for intensive surveillance	Highly promising; organ preservation remains an evolving strategy	[175,176]
Initially unresectable metastatic CRC	Chemotherapy combined with anti-EGFR or anti-VEGF therapy	Convert liver- or lung-limited disease to resectability	RAS/*BRAF* status, primary tumor sidedness, metastatic distribution, depth of response, organ reserve	Established in carefully selected patients	[178,189]
HCC	Locoregional and selected systemic therapies	Downstage or bridge patients to resection or liver transplantation	Tumor burden, radiological response, AFP dynamics, liver function, transplant eligibility	Locoregional strategies established; systemic and ICI-based approaches emerging	[190]
BTC and PDAC	Induction or biomarker-directed treatment	Enable secondary resection in exceptional responders	Durable disease control, anatomical feasibility, absence of progression, operative risk	Investigational and not routine	[186,187,191]
Surgery after anti-VEGF therapy	Planned interruption of treatment	Reduce wound-healing, bleeding, and anastomotic complications	Agent half-life, procedure extent, comorbidities, postoperative recovery	Requires individualized perioperative scheduling	[192,193]
Surgery after immunotherapy	Resection after neoadjuvant, perioperative, or conversion treatment	Allow safe surgery after systemic response	Active irAEs, organ function, corticosteroid exposure, timing of treatment, operative complexity	Generally feasible but context-dependent	[173]

**Table 4:** Indication-level FDA and EMA authorization status and Polish reimbursement-list status of selected biological and molecularly targeted therapies in gastrointestinal cancers as of 1 January 2026.

GI Cancer Indication	Drug/Regimen	Biomarker/Diagnostic Requirement	Disease Stage; Treatment Setting/Prior Therapy	FDA Status and Indication	EMA Status and Indication	Polish Reimbursement-List Status	References
Esophageal carcinoma/GEJ carcinoma	Pembrolizumab + platinum/fluoropyrimidine	FDA: no mandatory PD-L1 threshold in cited approval; EMA: PD-L1 CPS ≥ 10	Locally advanced unresectable or metastatic; First-line; not candidate for surgery or definitive chemoradiation	Authorized. Combination with platinum- and fluoropyrimidine-based chemotherapy	Authorized. First-line locally advanced unresectable/metastatic esophageal carcinoma; PD-L1 CPS ≥ 10	Listed for specific upper-GI indications. Programme/catalogue: B.58. Sheet B row 526 lists pembrolizumab under B.58 (also other programmes). Exact B.58 eligibility remains programme-specific	[209,210,211]
HER2-negative gastric/GEJ adenocarcinoma	Pembrolizumab + fluoropyrimidine/platinum	HER2-negative; EMA additionally PD-L1 CPS ≥ 1	Locally advanced unresectable or metastatic; First-line	Authorized. First-line HER2-negative gastric/GEJ adenocarcinoma; cited FDA approval did not impose a PD-L1 cutoff	Authorized. First-line; HER2-negative and PD-L1 CPS ≥ 1	Listed for specific upper-GI indications. Programme/catalogue: B.58. Sheet B row 526: B.58. Exact programme eligibility must be read from B.58	[112,210,211]
HER2-positive gastric/GEJ adenocarcinoma	Pembrolizumab + trastuzumab + fluoropyrimidine/platinum	HER2-positive; PD-L1 CPS ≥ 1	Locally advanced unresectable or metastatic; First-line	Authorized. Adults with HER2-positive disease and PD-L1 CPS ≥ 1	Authorized. HER2-positive and PD-L1 CPS ≥ 1	Listed for specific upper-GI indications. Programme/catalogue: B.58. Sheet B row 526: pembrolizumab B.58; trastuzumab is separately listed in chemotherapy catalogue C.86.a-c	[210,211,213]
Biliary tract cancer	Pembrolizumab + gemcitabine/cisplatin	No mandatory predictive biomarker	Locally advanced unresectable or metastatic; First-line/no prior systemic therapy for advanced disease	Authorized. Pembrolizumab + gemcitabine/cisplatin	Authorized. Pembrolizumab + gemcitabine/cisplatin, First-line	Not identified for BTC at cutoff. Sheet B row 526 does not list B.5. AOTMiT recommendation dated 16 December 2025 did not itself establish list inclusion effective 1 January 2026	[210,211,214]
Colorectal cancer	Pembrolizumab monotherapy	MSI-H or dMMR	Unresectable or metastatic; First-line	Authorized. First-line unresectable/metastatic MSI-H/dMMR CRC	Authorized. First-line metastatic MSI-H/dMMR CRC	Listed for CRC. Programme/catalogue: B.4. Sheet B row 526 lists B.4. Exact diagnostic and clinical criteria are programme-specific	[210,211,215]
MSI-H/dMMR gastric, small-intestine or biliary cancer/tumour-agnostic solid tumours	Pembrolizumab monotherapy	MSI-H or dMMR	Unresectable or metastatic/recurrent or advanced; After prior therapy; FDA requires progression and no satisfactory alternatives for tumour-agnostic indication	Authorized. Tumour-agnostic MSI-H/dMMR indication after progression and no satisfactory alternatives	Authorized. Gastric, small-intestine or biliary cancer after ≥1 prior therapy	Product listed, but indication-specific funding not established by product presence alone. Programme/catalogue: B.159 and other programmes. Sheet B row 526 lists multiple programmes, including B.159; programme wording must be checked before claiming reimbursement for a particular rare GI tumour	[210,211,216]
Gastric/GEJ cancer and esophageal adenocarcinoma	Nivolumab + fluoropyrimidine/platinum	FDA: no label biomarker threshold in cited approval record; EMA: PD-L1 CPS ≥ 5	Advanced or metastatic; First-line	Authorized. Advanced/metastatic gastric, GEJ and esophageal adenocarcinoma	Authorized. HER2-negative advanced/metastatic disease; PD-L1 CPS ≥ 5	Listed for upper-GI indications. Programme/catalogue: B.58. Sheet B rows 437–438 list B.58	[211,217,218]
Esophageal squamous cell carcinoma	Nivolumab + ipilimumab	FDA: no mandatory PD-L1 threshold in cited record; EMA: tumour-cell PD-L1 ≥ 1%	Unresectable advanced, recurrent or metastatic; First-line	Authorized. First-line unresectable advanced/metastatic ESCC	Authorized. First-line ESCC with tumour-cell PD-L1 ≥ 1%	Listed for upper-GI indications. Programme/catalogue: B.58. Sheet B rows 349–350 and 437–438 list both ipilimumab and nivolumab under B.58	[211,218,219]
Esophageal squamous cell carcinoma	Nivolumab + fluoropyrimidine/platinum	FDA: no mandatory PD-L1 threshold in cited record; EMA: tumour-cell PD-L1 ≥ 1%	Unresectable advanced, recurrent or metastatic; First-line	Authorized. First-line unresectable advanced/metastatic ESCC	Authorized. First-line ESCC with tumour-cell PD-L1 ≥ 1%	Listed for upper-GI indications. Programme/catalogue: B.58. Sheet B rows 437–438: B.58	[211,218,219]
Esophageal squamous cell carcinoma	Nivolumab monotherapy	No mandatory predictive biomarker	Unresectable advanced, recurrent or metastatic; After prior fluoropyrimidine- and platinum-based chemotherapy	Authorized. After prior fluoropyrimidine/platinum	Authorized. After prior fluoropyrimidine/platinum	Listed for upper-GI indications. Programme/catalogue: B.58. Sheet B rows 437–438: B.58	[211,218,220]
Esophageal or GEJ cancer	Nivolumab monotherapy	No mandatory predictive biomarker	Completely resected with residual pathologic disease; Adjuvant after neoadjuvant chemoradiotherapy and complete resection	Authorized. Adjuvant residual pathologic disease after neoadjuvant CRT	Authorized. Adjuvant residual pathologic disease after neoadjuvant CRT	Listed under B.58; exact adjuvant criteria require programme verification. Programme/catalogue: B.58. Sheet B rows 437–438: B.58	[211,217,218]
Colorectal cancer	Nivolumab + ipilimumab	MSI-H or dMMR	Unresectable or metastatic; All lines; FDA efficacy analysis included first-line; EMA includes first-line and after prior fluoropyrimidine combination	Authorized. Adults and patients ≥12 years with unresectable/metastatic MSI-H/dMMR CRC	Authorized. First-line and after prior fluoropyrimidine-based combination therapy	Listed for CRC. Programme/catalogue: B.4. Sheet B rows 349–350 and 437–438 list B.4	[211,218,221]
Hepatocellular carcinoma	Nivolumab + ipilimumab	No mandatory predictive biomarker	Unresectable or metastatic/advanced unresectable; First-line	Authorized. First-line unresectable/metastatic HCC	Authorized. First-line advanced/unresectable HCC	Not identified under B.5 at cutoff. Nivolumab and ipilimumab are listed in other programmes, but neither is assigned to B.5 in Sheet B rows 349–350 and 437–438	[211,218,222]
Hepatocellular carcinoma	Atezolizumab + bevacizumab	No mandatory predictive biomarker	Unresectable or metastatic/advanced unresectable; First-line/no prior systemic therapy	Authorized. Unresectable/metastatic HCC without prior systemic therapy	Authorized. Advanced/unresectable HCC without prior systemic therapy	Listed for HCC. Programme/catalogue: B.5; bevacizumab also C.82.a-d. Sheet B row 57 lists atezolizumab under B.5; Sheet C rows 52–59 list bevacizumab in C.82.a-d	[211,223,224]
Biliary tract cancer	Durvalumab + gemcitabine/cisplatin	No mandatory predictive biomarker	Unresectable or metastatic/locally advanced or metastatic; First-line	Authorized. Locally advanced/metastatic BTC	Authorized. First-line unresectable/metastatic BTC	Listed for BTC. Programme/catalogue: B.5. Sheet B row 171 lists durvalumab under B.5	[211,225,226]
Hepatocellular carcinoma	Durvalumab + tremelimumab	No mandatory predictive biomarker	Advanced or unresectable/unresectable; First-line	Authorized. Unresectable HCC (STRIDE regimen)	Authorized. First-line advanced/unresectable HCC	Combination not identified under B.5 at cutoff. Durvalumab is assigned to B.5 (Sheet B row 171), but tremelimumab is only B.6 (Sheet B row 707)	[211,226,227]
Gastric/GEJ adenocarcinoma	Durvalumab + FLOT, then durvalumab	No mandatory predictive biomarker	Resectable; FDA trial population Stage II-IVA; Neoadjuvant and adjuvant, followed by adjuvant durvalumab	Authorized. Approved 25 November 2025 for resectable gastric/GEJ adenocarcinoma	Not authorized in EU at cutoff. The relevant CHMP positive opinion was published 30 January 2026, after the 1 January 2026 cutoff; the current EPAR contains the later indication	Not identified for gastric/GEJ at cutoff. Durvalumab Sheet B row 171 is assigned to B.5/B.6/B.148, not B.58	[211,226,228]
HER2-negative gastric/GEJ adenocarcinoma	Tislelizumab + platinum/fluoropyrimidine	HER2-negative; FDA: PD-L1 ≥ 1; EMA: PD-L1 TAP ≥ 5%	Locally advanced unresectable or metastatic; First-line	Authorized. First-line unresectable/metastatic HER2-negative gastric/GEJ adenocarcinoma with PD-L1 ≥ 1	Authorized. HER2-negative; PD-L1 TAP ≥ 5%	Listed for upper-GI indications. Programme/catalogue: B.58. Sheet B row 672 lists B.58	[211,229,230]
Esophageal squamous cell carcinoma	Tislelizumab + platinum chemotherapy	FDA: PD-L1 ≥ 1; EMA: PD-L1 TAP ≥ 5%	Unresectable locally advanced or metastatic; First-line	Authorized. First-line unresectable/metastatic ESCC with PD-L1 ≥ 1, in combination with platinum-containing chemotherapy	Authorized. PD-L1 TAP ≥ 5%	Listed for upper-GI indications. Programme/catalogue: B.58. Sheet B row 672 lists B.58	[211,230,231]
Esophageal squamous cell carcinoma	Tislelizumab monotherapy	No mandatory predictive biomarker in EMA indication	Unresectable locally advanced or metastatic; After prior platinum-based chemotherapy	Authorized. Monotherapy after prior systemic chemotherapy that did not include a PD-(L)1 inhibitor	Authorized. After prior platinum-based chemotherapy	Listed for upper-GI indications. Programme/catalogue: B.58. Sheet B row 672 lists B.58	[211,230,231]
Gastric/GEJ adenocarcinoma	Trastuzumab + capecitabine or 5-FU/cisplatin	HER2 positive: IHC3+ or IHC2+ with confirmatory ISH in EMA label	Metastatic; No prior anticancer treatment for metastatic disease	Authorized. HER2-overexpressing metastatic gastric/GEJ adenocarcinoma; no prior treatment for metastatic disease	Authorized. HER2-positive metastatic gastric/GEJ; no prior metastatic anticancer treatment	Listed in chemotherapy catalogue. Programme/catalogue: C.86.a-c. Sheet C rows 466–475 list IV trastuzumab products under C.86.a-c	[211,232,233]
Gastric/GEJ adenocarcinoma	Trastuzumab deruxtecan	HER2 positive	Locally advanced or metastatic/advanced; After prior trastuzumab-based regimen	Authorized. Locally advanced/metastatic HER2-positive gastric/GEJ after prior trastuzumab	Authorized. Advanced HER2-positive gastric/GEJ after prior trastuzumab	Listed for upper-GI indications. Programme/catalogue: B.58. Sheet B row 701 lists Enhertu under B.58	[211,234,235]
Solid tumours, including selected GI cancers	Trastuzumab deruxtecan	HER2 IHC3+	Unresectable or metastatic; After prior systemic treatment; no satisfactory alternatives	Authorized (accelerated). Tumour-agnostic HER2-positive IHC3+ solid tumours	Authorized. Unresectable/metastatic HER2-positive IHC3+ solid tumours after prior treatment/no satisfactory options	Product listed, but tumour-agnostic reimbursement not established by B.58 presence. Programme/catalogue: B.58 for selected upper-GI indication. Sheet B row 701 lists B.58 only; this does not prove funding for every tumour-agnostic GI use	[211,235,236]
Biliary tract cancer	Zanidatamab	HER2 positive, IHC3+	Unresectable locally advanced or metastatic; After ≥1 prior systemic line; FDA study required prior gemcitabine-containing regimen	Authorized (accelerated). Previously treated unresectable/metastatic HER2-positive IHC3+ BTC	Authorized (conditional). Previously treated unresectable locally advanced/metastatic HER2-positive IHC3+ BTC	Not identified on Polish list at cutoff. No zanidatamab/Ziihera entry identified in Sheets B or C of 81W	[211,237,238]
Gastric/GEJ adenocarcinoma	Zolbetuximab + fluoropyrimidine/platinum	CLDN18.2 positive by validated/approved assay; HER2 negative	Locally advanced unresectable or metastatic; First-line	Authorized. First-line CLDN18.2-positive, HER2-negative gastric/GEJ	Authorized. First-line CLDN18.2-positive, HER2-negative gastric/GEJ	Not identified on Polish list at cutoff. No zolbetuximab/Vyloy entry identified in Sheets B or C of 81W	[211,239,240]
Colorectal cancer	Bevacizumab + fluoropyrimidine-based chemotherapy	No validated predictive biomarker	Metastatic; Indication/regimen-specific systemic treatment	Authorized. Metastatic colorectal cancer in indication-specific fluoropyrimidine-based chemotherapy combinations; see current FDA labeling	Authorized. Metastatic colon/rectal cancer with fluoropyrimidine-based chemotherapy	Listed in chemotherapy catalogue. Programme/catalogue: C.82.a-d. Sheet C rows 52–59 list bevacizumab products under C.82.a-d	[211,241,242]
Gastric/GEJ adenocarcinoma	Ramucirumab + paclitaxel/monotherapy	No universal predictive biomarker	Advanced; After prior platinum- and fluoropyrimidine-containing chemotherapy; monotherapy if combination unsuitable	Authorized. Advanced/metastatic gastric or GEJ adenocarcinoma after prior fluoropyrimidine- or platinum-containing chemotherapy; monotherapy or with paclitaxel	Authorized. Combination with paclitaxel after prior platinum/fluoropyrimidine; monotherapy in specified patients	Listed for upper-GI indications. Programme/catalogue: B.58. Sheet B row 555 lists B.58	[211,243,244]
Colorectal cancer	Ramucirumab + FOLFIRI	No validated predictive biomarker	Metastatic; After prior bevacizumab-, oxaliplatin- and fluoropyrimidine-containing therapy	Authorized. Metastatic CRC with FOLFIRI after prior bevacizumab-, oxaliplatin- and fluoropyrimidine-containing therapy; see current FDA labeling	Authorized. mCRC after bevacizumab, oxaliplatin and fluoropyrimidine	Listed in chemotherapy catalogue. Programme/catalogue: C.106. Sheet C row 401 lists ramucirumab under C.106	[211,244,245]
Hepatocellular carcinoma	Ramucirumab monotherapy	AFP ≥400 ng/mL	Advanced or unresectable; After sorafenib	Authorized. HCC with AFP ≥ 400 ng/mL after sorafenib	Authorized. Advanced/unresectable HCC with AFP ≥ 400 ng/mL after sorafenib	Not identified under B.5 at cutoff. Ramucirumab is B.58 and C.106, not B.5, in the 81W list	[211,244,246]
=Colorectal cancer	Aflibercept + FOLFIRI	No validated predictive biomarker	Metastatic; After resistance/progression to oxaliplatin-containing regimen	Authorized. Ziv-aflibercept with FOLFIRI for mCRC resistant to or progressed after an oxaliplatin-containing regimen; see current FDA labeling	Authorized. mCRC resistant to or progressed after oxaliplatin-containing regimen, with FOLFIRI	Listed in chemotherapy catalogue. Programme/catalogue: C.110. Sheet C rows 20–21 list Zaltrap under C.110	[211,247,248]
Colorectal cancer	Cetuximab + chemotherapy/monotherapy	RAS wild type; EMA also describes EGFR expression in indication wording	Metastatic; First-line FOLFOX or irinotecan-based combinations; monotherapy after specified failures/intolerance	Authorized. KRAS wild-type, EGFR-expressing mCRC in indication-specific combinations or monotherapy; see current FDA labeling	Authorized. RAS-wild-type mCRC in indication-specific combinations or monotherapy	Listed in chemotherapy catalogue. Programme/catalogue: C.95.a-c. Sheet C rows 108–109 list Erbitux under C.95.a-c	[211,249,250]
Colorectal cancer	Panitumumab + chemotherapy/monotherapy	RAS wild type	Metastatic; First-line FOLFOX/FOLFIRI; second-line FOLFIRI in specified setting; monotherapy after failures	Authorized. RAS wild-type mCRC in indication-specific combinations or monotherapy; see current FDA labeling	Authorized. RAS-wild-type mCRC in specified treatment lines/regimens	Listed in chemotherapy catalogue. Programme/catalogue: C.94. Sheet C rows 338–339 list Vectibix under C.94	[211,251,252]
Colorectal cancer	Encorafenib + cetuximab	BRAF V600E	Metastatic; After prior systemic therapy	Authorized. Previously treated BRAF V600E mCRC	Authorized. BRAF V600E mCRC after prior systemic therapy	Not identified for CRC at cutoff. Sheet B rows 217–218 list Braftovi only under B.59, not B.4	[211,253,254]
Colorectal cancer	Encorafenib + cetuximab + mFOLFOX6	BRAF V600E detected by FDA-approved test	Metastatic; First-line/treatment-naive population	Authorized (accelerated). FDA accelerated approval dated 20 December 2024	Not authorized in EU by cutoff; current EPAR includes a later first-line expansion. The first-line extension received a CHMP positive recommendation in May 2026, after the cutoff; it was not EU-authorized on 1 January 2026	Not identified for CRC at cutoff. Braftovi only B.59 in Sheet B rows 217–218	[211,255,256]
Colorectal cancer	Adagrasib + cetuximab	KRAS G12C by FDA-approved test	Locally advanced or metastatic; After fluoropyrimidine-, oxaliplatin- and irinotecan-based chemotherapy	Authorized (accelerated). FDA accelerated approval 21 June 2024	Not authorized for CRC at cutoff. Krazati EU authorization concerned NSCLC	Not identified on Polish list for CRC. No adagrasib/Krazati entry identified in Sheets B or C of 81W	[211,257,258]
Colorectal cancer	Sotorasib + panitumumab	KRAS G12C by FDA-approved test	Metastatic; After fluoropyrimidine-, oxaliplatin- and irinotecan-based chemotherapy	Authorized. FDA approval 16 January 2025	Not authorized for CRC at cutoff. Lumykras EU authorization concerned NSCLC	Not identified for CRC; product listed only in lung programme. Programme/catalogue: B.6 only. Sheet B rows 639–640 list Lumykras under B.6, not B.4	[211,259,260]
Cholangiocarcinoma	Pemigatinib monotherapy	FGFR2 fusion or rearrangement	Locally advanced or metastatic; Progression after ≥1 prior systemic line	Authorized (accelerated). Previously treated unresectable locally advanced/metastatic disease; FDA-approved test	Authorized (conditional). Progression after ≥1 prior systemic line	Not identified at cutoff. No pemigatinib/Pemazyre entry identified in Sheets B or C of 81W	[211,261,262]
Cholangiocarcinoma (FDA: intrahepatic)	Futibatinib monotherapy	FGFR2 fusion or rearrangement	Locally advanced or metastatic; After ≥1 prior systemic line/previously treated	Authorized (accelerated). Previously treated unresectable locally advanced/metastatic intrahepatic cholangiocarcinoma	Authorized (conditional). Locally advanced/metastatic cholangiocarcinoma after ≥1 prior systemic line	Not identified at cutoff. No futibatinib/Lytgobi entry identified in Sheets B or C of 81W	[211,263,264]
Cholangiocarcinoma	Ivosidenib monotherapy	FDA: susceptible IDH1 mutation by approved test; EMA: IDH1 R132 mutation	Locally advanced or metastatic; After ≥1 prior systemic line/previously treated	Authorized. Previously treated IDH1-mutated locally advanced/metastatic cholangiocarcinoma	Authorized. IDH1 R132; after ≥1 prior systemic line	Not identified for BTC; listed only in AML programme. Programme/catalogue: B.114 only. Sheet B row 368 lists Tibsovo under B.114, not B.5	[211,265,266]
Pancreatic adenocarcinoma	Olaparib monotherapy	Germline BRCA1/2 mutation	Metastatic; Maintenance; no progression after minimum 16 weeks of first-line platinum-based chemotherapy	Authorized. Maintenance gBRCAm metastatic pancreatic adenocarcinoma; FDA-approved companion diagnostic	Authorized. Maintenance germline BRCA1/2-mutated metastatic pancreatic adenocarcinoma after ≥16 weeks of first-line platinum	Listed for pancreatic cancer. Programme/catalogue: B.85. Sheet B rows 459–460 list Lynparza under B.85	[211,267,268]
NTRK fusion-positive solid tumours, including rare GI cancers	Larotrectinib monotherapy	Oncogenic NTRK gene fusion; FDA label excludes known acquired resistance mutation	Locally advanced, metastatic, or surgery likely to cause severe morbidity; No satisfactory alternatives/progressed after treatment (FDA wording)	Authorized. Tumour-agnostic NTRK fusion indication	Authorized (conditional). NTRK fusion; locally advanced/metastatic/severe-morbidity surgery; no satisfactory options	Listed for NTRK fusion solid tumours. Programme/catalogue: B.144. Sheet B rows 379–381 list Vitrakvi under B.144	[211,269,270]
NTRK fusion-positive solid tumours, including rare GI cancers	Entrectinib monotherapy	NTRK gene fusion; EMA requires no prior NTRK inhibitor	Locally advanced, metastatic, or surgery likely to cause severe morbidity; Progressed after treatment or no satisfactory alternatives	Authorized (accelerated). Tumour-agnostic NTRK fusion indication	Authorized (conditional). NTRK fusion; no prior NTRK inhibitor; no satisfactory options	Listed for NTRK fusion solid tumours. Programme/catalogue: B.144. Sheet B rows 229–230 list Rozlytrek under B.144 (also B.6 for ROS1 NSCLC)	[211,271,272]
dMMR recurrent or advanced solid tumours (FDA tumour-agnostic; potential GI context)	Dostarlimab monotherapy	dMMR by FDA-approved test	Recurrent or advanced; Progressed on/following prior treatment; no satisfactory alternatives	Authorized (accelerated). Tumour-agnostic dMMR solid tumour indication	No routine localized rectal organ-preservation indication. EMA product authorization must not be presented as routine approval for localized rectal cancer organ preservation	Not identified for GI indication; listed in endometrial programme. Programme/catalogue: B.148 only. Sheet B row 166 lists Jemperli under B.148	[211,273,274]

References correspond to official FDA, EMA, or Polish Ministry of Health records. FDA and EMA indication wording is intentionally kept separate. Polish list presence refers to the official notice effective 1 January 2026 and does not by itself reproduce all eligibility criteria contained in the full text of each drug program or chemotherapy catalogue. Abbreviations: BTC, biliary tract cancer; CPS, combined positive score; CRC, colorectal cancer; dMMR, mismatch-repair deficient; ESCC, esophageal squamous-cell carcinoma; FDA, US Food and Drug Administration; GEJ, gastroesophageal junction; HCC, hepatocellular carcinoma; HER2, human epidermal growth factor receptor 2; IHC, immunohistochemistry; ISH, in situ hybridization; mCRC, metastatic colorectal cancer; MSI-H, microsatellite instability-high; NTRK, neurotrophic tyrosine receptor kinase; PD-L1, programmed death-ligand 1; RAS WT, RAS wild type.

**Table 5:** Emerging technologies and potential applications in precision oncology for GI cancers.

Approach	Principal Information Generated	Potential Clinical Application	Main Advantages	Current Limitations	References
Genomic profiling	Mutations, amplifications, deletions, copy-number changes, and gene fusions	Identification of actionable alterations and eligibility for targeted or tumor-agnostic therapy	Established analytical platforms; direct link to several approved therapies	Does not fully reflect gene expression, pathway activity, or cellular context	[275,276]
Transcriptomics, proteomics, and metabolomics	Gene-expression programs, protein abundance, signaling activity, and metabolic states	Molecular classification, biomarker discovery, and identification of resistance pathways	Provides functional information beyond DNA alterations	Platform variability, complex data integration, high tissue and computational requirements	[275,276]
Single-cell sequencing	Cell-specific transcriptional or epigenetic states	Identification of malignant, immune, and stromal subpopulations associated with progression or treatment response	Resolves intratumoral heterogeneity obscured by bulk analysis	Loss of spatial context, cell-recovery bias, cost, and limited clinical standardization	[278,279]
Spatial transcriptomics and proteomics	Molecular profiles linked to anatomical tissue location	Identification of immune-excluded niches, stromal barriers, invasive fronts, and spatial predictors of response	Preserves tissue architecture and cellular interactions	High cost, platform heterogeneity, complex analysis, lack of validated clinical thresholds	[277,278]
Baseline ctDNA profiling	Tumor-derived genomic alterations detected in plasma	Molecular testing when tissue is insufficient and complementary assessment of metastatic heterogeneity	Minimally invasive and potentially representative of several disease sites	Low sensitivity in low-volume or low-shedding disease; clonal hematopoiesis	[280,281,282]
Postoperative ctDNA assessment	Molecular residual disease	Recurrence-risk stratification and potential guidance of adjuvant therapy	May detect residual disease before radiological recurrence	Optimal assay and sampling window remain uncertain; strongest evidence currently in CRC	[283,284,285]
Serial ctDNA monitoring	Changes in tumor burden and clonal composition over time	Monitoring response, detecting progression, and identifying acquired resistance	Enables repeated, minimally invasive molecular reassessment	A molecular change does not always indicate when or how treatment should be modified	[280,281,284,286]
Adaptive molecular profiling	Newly acquired or disappearing actionable alterations	Treatment switching, escalation, de-escalation, or rechallenge	Reflects dynamic tumor evolution rather than baseline status alone	Limited prospective evidence, cost, access, and lack of standardized intervention thresholds	[280,281,284]

Abbreviations: CRC, colorectal cancer; ctDNA, circulating tumor DNA; GC, gastric cancer; GI, gastrointestinal; MRD, molecular residual disease.

## Data Availability

No new data were generated or analyzed in this study. Data sharing is not applicable to this article.

## Abbreviations

The following abbreviations are used in this manuscript:

Abbreviation	Full term
ADC	Antibody–drug conjugate
ADCC	Antibody-dependent cellular cytotoxicity
ADCP	Antibody-dependent cellular phagocytosis
AFP	Alpha-fetoprotein
APC	Antigen-presenting cell
BRAF	B-Raf proto-oncogene
BRCA	Breast cancer susceptibility gene
BTC	Biliary tract cancer
CAR-T	Chimeric antigen receptor T cell
CCA	Cholangiocarcinoma
CDC	Complement-dependent cytotoxicity
CD	Cluster of differentiation
CLDN18.2	Claudin 18.2
CPS	Combined positive score
CRC	Colorectal cancer
CTLA-4	Cytotoxic T-lymphocyte-associated protein 4
dMMR	Deficient mismatch repair
EGFR	Epidermal growth factor receptor
EMA	European Medicines Agency
ERBB2	Erb-b2 receptor tyrosine kinase 2
ESCC	Esophageal squamous cell carcinoma
ESMO	European Society for Medical Oncology
FDA	United States Food and Drug Administration
FGF	Fibroblast growth factor
FGFR2	Fibroblast growth factor receptor 2
FGFR2b	Fibroblast growth factor receptor 2b
FISH	Fluorescence in situ hybridization
GC	Gastric cancer
GEJ	Gastroesophageal junction
GI	Gastrointestinal
GIST	Gastrointestinal stromal tumor
GPC3	Glypican-3
HCC	Hepatocellular carcinoma
HER2	Human epidermal growth factor receptor 2
HPV	Human papillomavirus
IDH1	Isocitrate dehydrogenase 1
IDH2	Isocitrate dehydrogenase 2
IHC	Immunohistochemistry
ISH	In situ hybridization
KIT	KIT proto-oncogene receptor tyrosine kinase
mAb	Monoclonal antibody
MMR	Mismatch repair
MSI	Microsatellite instability
MSI-H	Microsatellite instability-high
MSS	Microsatellite stable
NGS	Next-generation sequencing
NK	Natural killer
NTRK	Neurotrophic tyrosine receptor kinase
PALB2	Partner and localizer of BRCA2
PD-1	Programmed cell death protein 1
PD-L1	Programmed death-ligand 1
PDGFRA	Platelet-derived growth factor receptor alpha
PlGF	Placental growth factor
PRRT	Peptide receptor radionuclide therapy
RAS	Rat sarcoma viral oncogene homolog
SSTR	Somatostatin receptor
T-DXd	Trastuzumab deruxtecan
TKI	Tyrosine kinase inhibitor
TLA	Three-letter acronym
TRK	Tropomyosin receptor kinase
VEGF	Vascular endothelial growth factor
VEGFR	Vascular endothelial growth factor receptor
WT	Wild-type
